# *AvrPm2* encodes an RNase‐like avirulence effector which is conserved in the two different specialized forms of wheat and rye powdery mildew fungus

**DOI:** 10.1111/nph.14372

**Published:** 2016-12-09

**Authors:** Coraline R. Praz, Salim Bourras, Fansong Zeng, Javier Sánchez‐Martín, Fabrizio Menardo, Minfeng Xue, Lijun Yang, Stefan Roffler, Rainer Böni, Gerard Herren, Kaitlin E. McNally, Roi Ben‐David, Francis Parlange, Simone Oberhaensli, Simon Flückiger, Luisa K. Schäfer, Thomas Wicker, Dazhao Yu, Beat Keller

**Affiliations:** ^1^ Department of Plant and Microbial BiologyUniversity of ZürichZürich8008Switzerland; ^2^ Institute of Plant Protection and Soil ScienceHubei Academy of Agricultural SciencesWuhan430064China; ^3^ Ministry of Agriculture Key Laboratory of Integrated Pest Management in Crops in Central ChinaWuhan430064China; ^4^ College of Life ScienceWuhan UniversityWuhan430072China; ^5^ Institute of Plant ScienceARO‐Volcani CenterBet Dagan50250Israel

**Keywords:** avirulence gene, *Blumeria graminis*, *Pm2*, powdery mildew, RNAse‐like, wheat

## Abstract

- There is a large diversity of genetically defined resistance genes in bread wheat against the powdery mildew pathogen *Blumeria graminis* (*B. g*.) f. sp.* tritici*. Many confer race‐specific resistance to this pathogen, but until now only the mildew avirulence gene *AvrPm3* ^*a2/f2*^ that is recognized by *Pm3a/f* was known molecularly.
- We performed map‐based cloning and genome‐wide association studies to isolate a candidate for the mildew avirulence gene *AvrPm2*. We then used transient expression assays in *Nicotiana benthamiana* to demonstrate specific and strong recognition of *AvrPm2* by *Pm2*.
- The virulent *AvrPm2* allele arose from a conserved 12 kb deletion, while there is no protein sequence diversity in the gene pool of avirulent *B. g. tritici* isolates. We found one polymorphic *AvrPm2* allele in *B. g. triticale* and one orthologue in *B. g. secalis* and both are recognized by *Pm2*. *AvrPm2* belongs to a small gene family encoding structurally conserved RNase‐like effectors, including *Avr*_*a13*_ from *B. g. hordei*, the cognate *Avr* of the barley resistance gene *Mla13*.
- These results demonstrate the conservation of functional avirulence genes in two cereal powdery mildews specialized on different hosts, thus providing a possible explanation for successful introgression of resistance genes from rye or other grass relatives to wheat.

## Abstract 2

See also the Commentary on this article by Spanu, **213:** 969–971.

## Introduction

Obligate biotrophic fungi are major pathogens of cultivated crops. They establish a long‐term feeding relationship with a living host throughout their life cycle, suggesting highly effective and long‐lasting host defence suppression and physiological reprogramming (Kemen & Jones, 2012). Two of the most agronomically important groups of obligate biotrophic fungal pathogens are basidiomycete rusts and ascomycete powdery mildews. Powdery mildews are pathogens that infect monocot and dicot species including important crops such as cereals, grape and tomato. Cereal mildews are grouped into one species, *Blumeria graminis* (*B. g*.), that is divided in different *formae speciales* corresponding to pathogens adapted to a specific host species (Schulze‐Lefert & Panstruga, 2011). Of these, five grow on cultivated cereals: *B. g. tritici* on wheat, *B. g. hordei* on barley, *B. g. secalis* on rye, *B. g. avenae* on oat and the newly identified *B. g. triticale* form that arose from a hybridization between *B. g. tritici* and *B. g. secalis* and grows on triticale, bread wheat and durum wheat (Troch *et al*., 2014; Menardo *et al*., 2016).

Resistance against obligate biotrophic pathogens is commonly mediated by *R* genes encoding intracellular immune receptors mostly belonging to the conserved protein family of nucleotide‐binding, leucine‐rich repeat receptor (NLR) proteins (Dodds & Rathjen, 2010). Forty‐five genetic loci have been identified in cereal crops that confer agronomically effective resistance against the powdery mildew fungus *B. graminis*, but only a few of them have been cloned (Yahiaoui *et al*., 2006; Bhullar *et al*., 2009; Seeholzer *et al*., 2010; Hurni *et al*., 2013; Sánchez‐Martín *et al*., 2016). Mildew resistance genes have been introgressed from rye and wild grasses into wheat, indicating high conservation of effector recognition in different cereal species despite host specialization on the pathogen side. Two of the best characterized *R* genes in cereals are *Mla* in barley and *Pm3* in wheat, both forming allelic series (Yahiaoui *et al*., 2006; Bhullar *et al*., 2009; Seeholzer *et al*., 2010). R proteins interact directly or indirectly with cognate pathogen avirulence factors (AVRs), and upon recognition many induce a hypersensitive response (HR), a form of programmed cell death that is particularly effective against obligate biotrophs (Moffett *et al*., 2002). Domain swap studies have shown that the LRR domain is a major determinant of AVR recognition specificity, and a few amino acid differences in this domain can result in distinctly different resistance spectra (Brunner *et al*., 2010; Ravensdale *et al*., 2011).

There are few *Avr* genes isolated from obligate biotrophic fungi and they typically encode for small proteins with a predicted signal peptide and no homology to any known protein function (Ravensdale *et al*., 2011; Bourras *et al*., 2015; Lu *et al*., 2016). Two exceptions are AVR_a10_ and AVR_k1_ from barley powdery mildew: they are encoded within long interspersed nuclear elements (LINE) retrotransposons (Ridout *et al*., 2006; Amselem *et al*., 2015). Most of the *Avr* genes cloned from obligate biotrophic fungi have been isolated from the flax rust fungus *Melampsora lini* (*AvrL567*,* AvrM*,* AvrP123* and *AvrP4*) and control race‐specific recognition by the *L*,* M* and *P* allelic series of *R* genes in flax (Ravensdale *et al*., 2011). Amino acid polymorphisms between *Avr* variants are associated with differences in recognition specificity. For example, Dodds *et al*. (2006) showed that seven out of 12 variants of *AvrL567* are differentially recognized by the *L5*,* L6* or *L7* allele, while the other five are not recognized. In powdery mildews, the first *Avr* coding for a typical effector protein has been cloned recently (Bourras *et al*., 2015). *AvrPm3* ^*a2/f2*^ is highly expressed at the stage of haustorium formation and confers dual recognition specificity towards the wheat *Pm3a* and *Pm3f* resistance gene alleles. In contrast to flax rust where *Avr* variants with different allelic specificities can be encoded within one cluster (e.g. *AvrL567* variants A, B, F and L; Ellis *et al*., 2007), there is only one *AvrPm3* ^*a2/f2*^ gene in the mildew genome, and the closest effector homologues are not recognized by any alleles of the *Pm3* resistance gene (Bourras *et al*., 2015). Thus, while the structural and molecular basis of race‐specific resistance to obligate biotrophic fungi is conserved on the plant side, there seem to be major differences in the way avirulence and gain of virulence are controlled on the pathogen side.

The powdery mildew genomes are some of the largest among fungi with an estimated size of 150–180 Mb, 90% of which corresponds to transposable element (TE) sequences (Spanu *et al*., 2010; Wicker *et al*., 2013). Contrasting with genome size, the gene content of powdery mildews is one of the lowest in fungi with *c*. 6500 genes, compared to an average of 11 000 in ascomycetes and 15 000 in basidiomycetes (Wicker *et al*., 2013; Mohanta & Bae, 2015). The mildew genomes encode a very large complement of candidate secreted effector proteins (CSEPs) that account for almost 10% of all predicted coding genes (Spanu *et al*., 2010; Pedersen *et al*., 2012; Wicker *et al*., 2013). Mildew effectors are predominantly expressed in the haustorium, and there is increasing molecular evidence of their direct implication in pathogen virulence (Zhang *et al*., 2012; Pliego *et al*., 2013; Schmidt *et al*., 2014; Ahmed *et al*., 2015). A functional screen of barley powdery mildew effectors using host‐induced gene silencing resulted in the identification of two CSEPs, BEC1054 (syn. CSEP0064) and BEC1011 (syn. CSEP0264) whose down‐regulation led to a reduction of 60 and 70%, respectively, in the ability of the mildew to form haustoria (Pliego *et al*., 2013). BEC1054 targets several barley proteins including a pathogen‐related‐5 protein isoform, suggesting a role in suppression of pathogen‐associated molecular pattern (PAMP)‐triggered immunity (PTI) (Pennington *et al*., 2016). Based on structural homologies to known fungal ribonucleases, BEC1054 and BEC1011 were classified as RNase‐like proteins, which make up the largest class of mildew CSEPs, accounting for almost 15% of all *B. g. hordei* effectors (Pedersen *et al*., 2012; Pliego *et al*., 2013). In *B. g. tritici*,* SvrPm3* ^*a1/f1*^ is a ribonuclease‐like effector involved in suppressing the *AvrPm3* ^*a2/f2*^ *–Pm3a/f* effector‐triggered immunity (ETI) (Bourras *et al*., 2015; Parlange *et al*., 2015). Together, these results suggest a central role of RNase‐like effectors in controlling mildew virulence and race specificity. Their exact mode of action is poorly understood, but a possible role as modulator of host immunity via interactions with host RNAs was proposed (Pedersen *et al*., 2012; Spanu, 2015).

The wheat resistance gene *Pm2* has been molecularly isolated by mutant chromosome sequencing (MutChromSeq) and encodes an NLR protein (Sánchez‐Martín *et al*., 2016). Here we report the cloning of *AvrPm2*, the cognate *Avr* of *Pm2*. We demonstrate specific recognition and strong induction of a typical hypersensitive cell‐death response in transient assays in *Nicotiana benthamiana*. We show that *AvrPm2* belongs to a ribonuclease‐like effector family that is conserved among cereal mildews and structurally different from *AvrPm3* ^*a2/f2*^. We also show that *Pm2* recognizes the close homologue *BgsE‐5845* from rye powdery mildew, indicating evolutionary conservation of this avirulence effector, and providing the first molecular explanation for functional *R* gene introgression in cereals.

## Materials and Methods

### Fungal crosses, plant material and virulence tests

*Blumeria graminis* isolates and the progeny from the cross between the Swiss isolate 96224 (mating‐type MAT1‐2) and the British isolate JIW2 (mating type MAT1‐1) were maintained in the haploid asexual phase on detached leaves of the susceptible wheat (*Triticum aestivum*) cultivar Kanzler on benzimidazole agar as described by Parlange *et al*. (2011). The near isogenic lines Ulka/8*Chancellor, Federation*4/Ulka and CI12632/8*Chancellor carrying the *Pm2* resistance gene were used for phenotyping the progeny (Parlange *et al*., 2015; Sánchez‐Martín *et al*., 2016).

### DNA/RNA isolation and construction of plasmid vectors

High molecular weight DNA of fungal isolates and RNA samples from infected leaves were extracted as previously described (Bourras *et al*., 2015). RNA samples were extracted from infected leaf material using the Qiagen miRNeasy Mini Kit (Qiagen) according to the manufacturer's instructions. RNA quality was checked by gel electrophoresis and based on the 260 : 280 ratios measured with a spectrophotometer (Nanodrop, Thermo Fisher Scientific, Waltham, MA, USA). The 260 : 280 ratios are available in Supporting Information Table S1. Full‐length cDNA was prepared using the SuperScript III RT kit (Life Technologies) according to the manufacturer's instructions. Molecular cloning of powdery mildew and wheat genes into Gateway‐compatible entry vector was performed using the pENTR/D‐TOPO Cloning Kit (Life Technologies) according to the manufacturer's instructions (list of primers in Table S2). Recombination to the binary vector pIPKb004 was performed as described by Stirnweis *et al*. (2014). RACE‐PCR cDNA was prepared with the SMARTer RACE cDNA kit (Takara Bio, Shiga, Japan) according to manufacturer's instructions.

### Genetic mapping

Genetic maps were constructed with Kompetitive Allele‐Specific PCR (KASP) markers as described by Bourras *et al*. (2015). We produced a consensus linkage group containing the *AvrPm2* locus as described by Bourras *et al*. (2015). For fine mapping, additional markers were designed on single nucleotide polymorphisms (SNPs) predicted between the parental sequences and genotyped by direct Sanger sequencing of PCR amplicons, or by using a cleaved amplified polymorphic sequence (CAPS) marker. To genetically anchor the genes identified by collinearity between wheat and barley powdery mildew to our genetic map, SNPs located in or near those genes were scored by direct Sanger sequencing of PCR amplicons. To validate co‐segregation of *BgtE‐5845* with *AvrPm2*, presence/absence markers were designed on and near the gene and tested by PCR. All markers/primers used for genetic mapping are listed in Table S3.

### BAC selection, sequencing and *in silico* annotation of the *AvrPm2* locus

Bacterial artificial chromosomes (BACs) spanning the *AvrPm2* interval were selected from the BAC library (Parlange *et al*., 2011) based on their position in two different assemblies, confirmed by PCR, sequenced using Illumina MiSeq technology (San Diego, CA, USA), and assembled. Genes and TEs were then annotated manually (Table S4; Methods S1). The *AvrPm2* region is deposited in the GenBank/EMBL library (accession number KX765276). To identify the *AvrPm2* variant in the virulent parent JIW2, genome sequencing reads were mapped on the assembly of the *AvrPm2* locus using CLC genomics software with the following parameters: mismatch cost = 2, insertion cost = 2, length fraction = 0.9, similarity fraction = 0.95. Physical markers were designed every 500 bp in a 20 kb region around *AvrPm2* and amplification products were obtained from three isolates. All PCR products were checked by gel electrophoresis, purified and sequenced. The physical markers used to estimate the size of the deletion are listed in Table S5.

### Genome‐wide association study (GWAS)

A set of 22 Chinese isolates were resequenced (Table S6; Methods S2). We mapped Illumina reads from the genomes of 60 isolates (Table S7) on the *B. g. tritici* reference (Wicker *et al*., 2013) using Bowtie2 (Langmead & Salzberg, 2012) with the following parameters: – score‐min L, −0.6, −0.25. We used SAMtools 0.1.19 (Li *et al*., 2009) to convert formats and collect genotype information at every polymorphic position. Finally, we used bcftools (Danecek *et al*., 2011) to generate a VCF file that was parsed with in‐house Perl scripts. We considered as high‐confidence SNPs all positions with a minimum mapping score of 20, a minimum coverage of 8×, a minimum frequency of the alternative call of 0.9 and < 5 missing genotypes. The GWAS was performed with the R package Gapit (Lipka *et al*., 2012). We used vcftools to calculate *AvrPm2* locus coverage for every isolate using sliding windows of size 1000 bp with a 1000 bp step. All genomic sequences used for this study are available at the Sequence Read Archive (SRA) under accession number SRP062198.

### Transient protein expression assays in *N. benthamiana* and HR measurements

Transient expression by agroinfiltration in *N. benthamiana* was performed as described in Bourras *et al*. (2015). HR was visually inspected 5 d after agroinfiltration and revealed by fluorescence scanning using the Fusion FX Imaging System (Vilbert Lourmat, Eberhardzell, Germany) with the following pre‐settings: lighting, blue epi‐illumination; excitation, spectra blue 470 nm; emission, F‐535 Y2‐filter; aperture, 0.84. Fluorescence intensity was quantified using the ImageJ2 image processing program (Schindelin *et al*., 2015). The measure of fluorescence is in integrated density, the product of the area infiltrated and the mean grey value. These fluorescence measurements were used to conduct an ANOVA followed by Tukey HSD *post‐hoc* tests using R. All gene sequences used in expression constructs for transient protein expression assays in *N. benthamiana* are available in Notes S1.

### qRT‐PCR experiments

Quantitative real‐time PCR (qRT‐PCR) ready cDNA was synthesized from 1.5 ?g total RNA using the iScript cDNA synthesis kit (Bio‐Rad) according to the manufacturer’s instructions. Glyceraldehyde 3‐phosphate dehydrogenase (Gapdh) and Actin (Act1) genes were used as internal controls as described by Bourras *et al*. (2015). Gene expression was normalized to that of Gapdh. Target and reference gene fragments were amplified using the KAPA SYBR FAST qPCR kit (Kapa Biosystems, Wilmington, MA, USA) and the CFX96 real‐time PCR detection system (Bio‐Rad) according to the manufacturer's instructions. Gene expression analysis was performed using the Bio‐Rad real‐time PCR CFX Manager software v.3.5, according to the manufacturer's instructions (http://www.bio-rad.com/en-ch/product/cfx-manager-software). Additional information about qRT‐PCR experiments methodology is available in Methods S3. qRT‐PCR primers for *AvrPm2* are listed in Table S2 and the primer efficiency curve is depicted in Fig. S1.

### Phylogenetic analysis

We performed a *de novo* annotation of the *B. g. tritici* genome, redefined the prediction of effector families and identified the *AvrPm2* family. We also identified haplotypes of *AvrPm2* in other *formae speciales* (Methods S4). Multiple alignments using gene sequences coding for the full protein as well as truncated sequences coding for the mature peptide were performed with Muscle 1:3.8.31‐1 (Edgar, 2004) and retrotranslated with TranslatorX (Abascal *et al*., 2010). Raxml v8.2.8 (Stamatakis, 2006) was used to find the maximum likelihood tree using GTR + GAMMA model and bootstrap support was computed with 100 replications. To test for positive selection, we estimated the likelihood of the maximum likelihood tree under the M7 and M8 models (Yang *et al*., 2000) with Paml 4.8 (Yang, 2007) and subsequently used the likelihood ratio test. The sequences of the *AvrPm2* effector family used for phylogenetic analyses are available in Notes S2 (coding sequences) and Notes S3 (protein sequences).

## Results

### Avirulence towards *Pm2* is controlled by a single genetic locus in wheat powdery mildew

**Figure 1:**
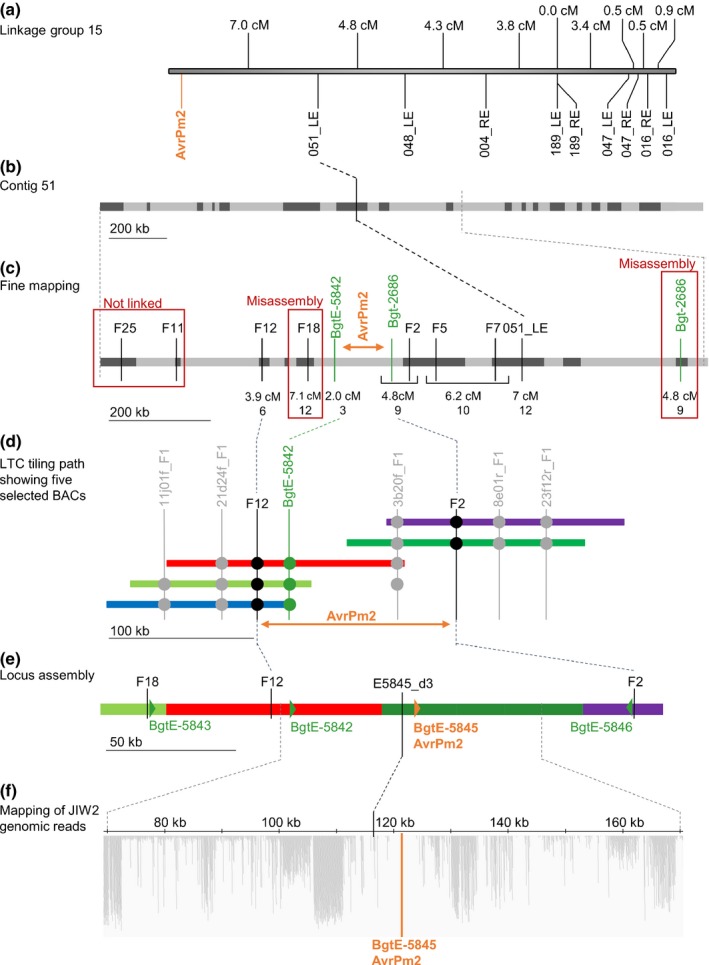
Genetic and physical mapping of *AvrPm2*. (a) Linkage group 15 containing the *AvrPm2* locus and the closest Kompetitive Allele‐Specific PCR (KASP) markers. Distances between markers are indicated in cM. (b) Contig 51 containing the *AvrPm2* linked marker 051_LE. Dark grey boxes represent regions for which sequences are available (Wicker *et al*., 2013) and light grey boxes are sequence gaps. (c) Fine mapping of the *AvrPm2* region. The most informative cleaved amplified polymorphic sequence (CAPS) marker and single nucleotide polymorphism (SNP) markers are indicated in black. Markers designed on the genes identified by the collinearity approach (Supporting Information Fig. S1; Notes S5) are depicted in green. Genetic distances between each marker and *AvrPm2* are indicated in cM and in number of recombinants with *AvrPm2*. Red frames indicate misassemblies in the genome sequence. The interval containing *AvrPm2* is indicated in orange. (d) Bacterial artificial chromosomes (BACs) minimal tiling path of the LTC scaffold spanning the *AvrPm2* region. The markers designed on BAC end sequences and used to validate the BAC overlap are indicated with vertical grey lines and the presence of specific PCR amplifications is indicated with grey dots. The most informative markers are indicated with black and green vertical lines, and presence of specific PCR amplifications with black and green dots, respectively. The *AvrPm2* interval is indicated in orange. (e) Schematic representation of the assembly of the *AvrPm2* region created from four sequenced BACs. The positions of the informative markers are indicated in black. The *AvrPm2* candidate gene interval is indicated in orange and other genes in green. (f) Visual representation of the mapping of genomic sequencing reads from the virulent parent JIW2 on the *AvrPm2* locus assembly. Here, 50 kb up‐ and downstream of the gene *BgtE‐5845* is represented. The position of *BgtE‐5845* is represented with an orange vertical line and no reads map in and around *BgtE‐5845*, indicating a sequence deletion in the virulent parent JIW2.

To identify *AvrPm2* we used two different genetic approaches: high‐resolution genetic mapping in an F1 population and a genome wide association study using a collection of geographically distant powdery mildew isolates. We have previously produced a cross between the *Pm2* avirulent isolate 96224 and the *Pm2* virulent isolate JIW2 and generated a mapping population of 117 individuals (Parlange *et al*., 2015). This F1 haploid progeny segregated in a 1 : 1 phenotypic ratio of avirulence to virulence on a wheat genotype with the *Pm2* gene, indicating the presence of a single *AvrPm2* locus in the pathogen (Parlange *et al*., 2015). To map the genetic interval containing *AvrPm2*, we used 254 SNP markers that we have previously scored with KASP technology in the 117 progeny (He *et al*., 2014; Bourras *et al*., 2015). We generated a genetic map of 1275 cM that included 248 markers spread over 18 linkage groups (LGs) containing at least four markers and ranging in size from 10.3 to 173.1 cM (Table S8). *AvrPm2* mapped at the end of LG_15, at 7.0 cM south of marker M051_LE, indicating that the gene was genetically linked to contig 51 of the mildew genome assembly (Fig. 1a).

Analysis of contig 51 revealed that 1.52 (76.4%) of the predicted 2 Mb assembly consisted of sequence gaps of various length (Fig. 1b; Notes S4). To reduce the genetic interval containing *AvrPm2*, we developed a series of markers designed on all the SNPs scored between the parental genomic sequences (CAPS and SNP markers tested by sequencing). A second approach consisted of anchoring additional sequences to the fragmented contig 51 based on collinearity with contiguous scaffolds in the *B. g. hordei* genome, thus allowing the development of additional SNP markers (Notes S5). All markers were tested on the 117 progeny. Together, these approaches resulted in: essential improvements of sequence assembly and gene order in contig‐51 (Fig. S2); the development of 20 additional SNP markers; and the identification of new flanking markers (F2 and BgtE‐5842) defining a 6.8 cM genetic interval containing *AvrPm2* (Fig. 1c).

### The *AvrPm2* locus contains a cluster of effector gene homologues

To determine the full sequence of the *AvrPm2* locus, we sequenced the minimal tiling path of the BAC contigs spanning the 8.7 cM interval between markers F2 and F12 (Figs 1c, S3; Notes S4). The resulting sequence reads were assembled using the CLC genomics software, which resulted in a 217 kb contiguous sequence spanning the *AvrPm2* locus, a region that was originally estimated to be 250 kb (Figs 1e, S3; Notes S4). The sequence contained mainly TEs (> 80%) and a cluster of four homologous effector genes, *BgtE‐5842*,* BgtE‐5843*,* BgtE‐5845* and *BgtE‐*5846. To reduce the number of *AvrPm2* avirulence gene candidates we specifically searched for genes that are polymorphic between the two parental isolates, genetically co‐segregate with *AvrPm2*, and are expressed in the avirulent parent 96224. Two genes, *BgtE‐5843* and *BgtE‐5846*, are not expressed as determined by RNA‐Seq data (Wicker *et al*., 2013) and mapped outside of the genetic interval containing *AvrPm2* as defined by the flanking markers. *BgtE‐5842* was expressed in 96224 but mapped at three recombinants from *AvrPm2*, thus leaving only one possible candidate: *BgtE‐5845*.

To test whether *BgtE‐5845* cosegregates with *AvrPm2* in the F1 population, we first analysed sequence polymorphisms between the parental genomes by mapping the sequencing reads from the virulent parent JIW2 on the model assembly of the *AvrPm2* locus (Fig. 1f). Consistent with results from Wicker *et al*. (2013), we found that *BgtE‐5845* was located in a region of several kilobases that is deleted in the virulent parent JIW2. Based on PCR amplification of physical markers spanning a 20 kb interval in the model assembly, we estimated the size of the deletion in the JIW2 virulent parent to be at least 10 kb (Figs S4, S5). Therefore, we assessed the co‐segregation between *BgtE‐5845* and *AvrPm2* using two InDel markers, E5845Int1 and E5845Int2, designed in the coding sequence of *BgtE‐5845*, and two additional ones, E5845_100 and E5845_d3, designed 100 and 4.6 kb upstream of the gene, respectively (Figs 1f, S4). The four markers were tested on the parents and the 12 recombinant progeny defining the genetic interval between the markers flanking *AvrPm2*. The results indicated that the gene and up to 4.6 kb of upstream sequence are only present in 96224 and the *Pm2* avirulent recombinant progeny, thus indicating that *BgtE‐5845* co‐segregates with *AvrPm2*.

**Figure 2:**
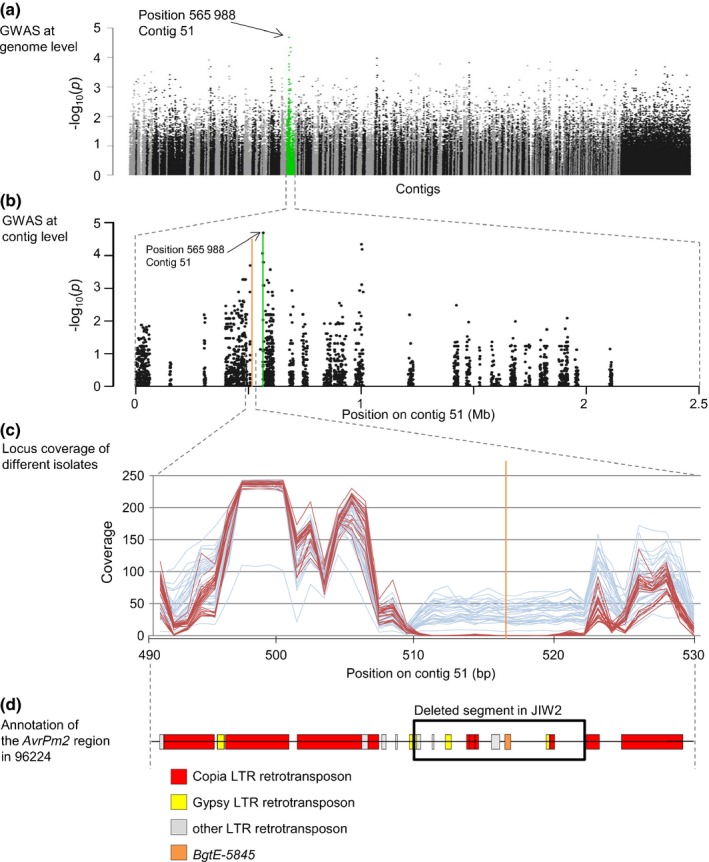
Genome wide association mapping of *AvrPm2* in a set of 41 *Blumeria graminis* (*B. g*.) *tritici* and 19 *B. g. triticale* isolates. (a) Genome‐wide association study (GWAS) results at the genome level. The −Log_10_ of the test *P* value of 424 219 single nucleotide polymorphisms (SNPs) after correction by λ is plotted against its physical contig position. Linkage groups are shown in different colours. Contig 51 containing the best correlated SNP is shown in green. The best correlated SNP is indicated with an arrow. (b) GWAS results at the contig level. The −Log_10_ of the test *P* value of the SNPs located on contig 51. The best correlated SNP is indicated with a vertical green line and the position of the gene *BgtE‐5845* with a vertical orange line. (c) The coverage value per 1000 bp window of a 40 kb interval around the position of *BgtE‐5845* (orange vertical line) is plotted. The blue lines indicate avirulent isolates and the red lines virulent isolates. In all virulent isolates, the coverage between positions 510 000 and 522 000 is < 5×, indicating a deletion of 12 kb in the virulent isolates. (d) Annotation of the region containing *AvrPm2* and the 12 kb deletion in the *Pm2* avirulent isolate 96224. The gene *BgtE‐5845* is indicated with an orange box. Other boxes indicate transposable elements. The deleted sequence in JIW2 is indicated with a black frame.

In our second approach to identify *AvrPm2*, we performed a GWAS using 60 cereal mildew genomes including 42 *B. g. tritici* (12 virulent and 30 virulent) and 18 *B. g. triticale* (15 virulent and three avirulent) isolates originating mainly from Switzerland, Israel and China (Wicker *et al*., 2013; Menardo *et al*., 2016; Table S7). Genetic association between sequence polymorphisms and differences in virulence/avirulence patterns on *Pm2* was assessed using the Genome Association and Prediction Integrated Tool (GAPIT; Lipka *et al*., 2012). We found the best correlated SNP at position 565 988 on contig 51, which is 35 kb from the closest predicted effector gene *BgtE‐5846*, and 50 kb from *BgtE‐5845* (Fig. 2a,b). As mentioned before, *BgtE‐5846* is not polymorphic between 96224 and JIW2, and RNA‐Seq mapping indicated it is not expressed in the avirulent parent. Manual inspection of the *AvrPm2* locus sequence in the 60 mildew genomes revealed that *BgtE‐5845* was present in all *Pm2* avirulent isolates and absent in all the virulent ones (Table S7). To identify the size of the deletion in these geographically diverse isolates, we analysed sequencing coverage over a 40 kb region around *BgtE‐5845*. We found that the coverage was similar among all isolates over the 40 kb region except for a segment of 12 kb where the average coverage was *c*. 45× in the avirulent isolates and lower than 5× in all the virulent ones, thus suggesting a 12 kb deletion that contained *BgtE‐5845* (Fig. 2c). We found that the sequence outside of the deletion consisted of high copy number long terminal repeat (LTR) retrotransposons of the superfamily *Copia* (family Ino), while the deleted segment contained mainly low copy number solo LTRs (Wicker *et al*., 2007), indicating a particular structure of the *AvrPm2* locus based on TE content (Fig. 2d).

In conclusion, two different approaches identified *BgtE‐5845* as the best and only candidate for *AvrPm2*, and that the virulent allele arose from a deletion in the virulent parent. *BgtE‐5845* encodes for a typical effector protein of 120 residues that is slightly shorter than AVRPM3^A2/F2^ (130 residues) (Bourras *et al*., 2015). The N‐terminal region of the native protein consists of a predicted 21 amino acid long signal peptide followed by a Y(x)xC motif commonly found in mildew CSEPs (SignalP 4.1; Petersen *et al*., 2011; Pedersen *et al*., 2012). The predicted mature protein consists of 99 residues and contains a C‐terminal cysteine that is predicted to form a disulphide bond with the N‐terminal cysteine in the Y(x)xC motif (Disulfind; Ceroni *et al*., 2006).

### Characterization of *Pm2*, the cognate *R* gene of *AvrPm2*

**Figure 3:**
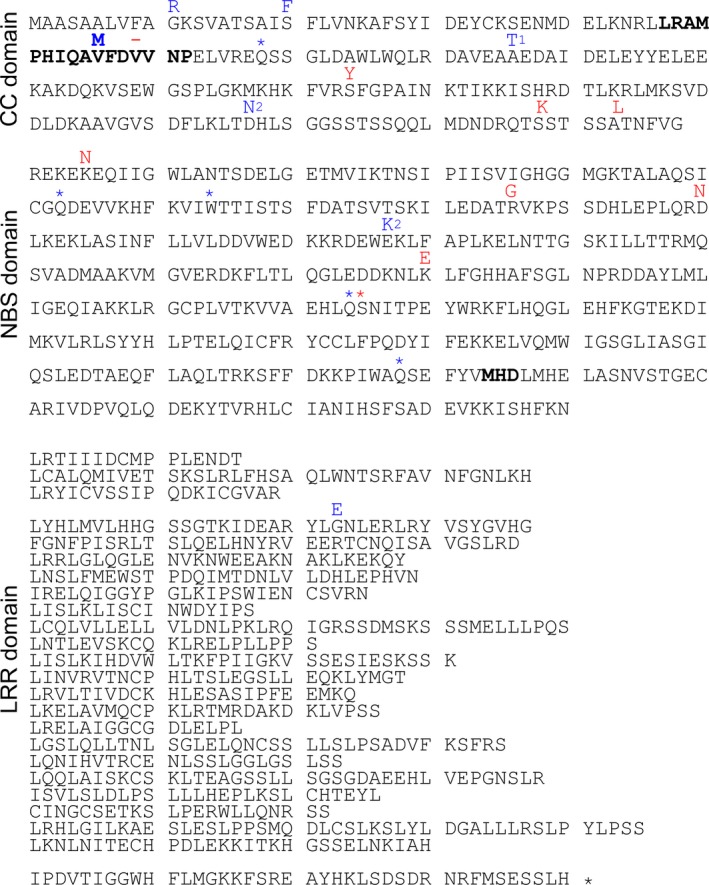
Amino acid sequence of the protein encoded by *Pm2*. The coiled‐coil (CC), nucleotide binding domain (NB) and leucine rich repeat (LRR) domains are indicated on the left. Polymorphisms present in the susceptible allele are represented in red until the premature stop codon at position 423 highlighted with a red star. The amino acids represented in blue show the mutations found in the EMS mutants described by Sánchez‐Martín *et al*. (2016). Residues marked with ‘1’ indicate mutations found in two independent mutants. Residues marked with ‘2’ indicate mutations found in the same mutant.

The wheat resistance gene *Pm2* was recently identified by MutChromSeq in the cultivar Federation*4/Ulka (Sánchez‐Martín *et al*., 2016). The structure of the gene consists of three exons (3730, 58 and 46 bp) and two introns (104 and 468 bp) coding for a protein of 1278 amino acids, slightly shorter than the well‐studied PM3 alleles (1415 amino acids) (Yahiaoui *et al*., 2004; Sánchez‐Martín *et al*., 2016). We analysed the predicted protein sequence for conserved domains and identified an N‐terminal coiled‐coil (CC) domain of 198 amino acids, a central nucleotide binding domain (NB) of 390 amino acids and 23 C‐terminal leucine rich repeats (LRRs) of various length (16–47 amino acids) (Fig. 3; Methods S5).

The susceptible *Pm2* allele in cultivar Chinese Spring encodes a truncated protein (422 amino acids) that arose from a 7 bp deletion introducing a frame shift and a premature stop codon. In addition, within the 422 amino acids encoded by the truncated version of the gene, we found 14 SNPs between susceptible and resistant *Pm2* alleles leading to 7 amino acid polymorphisms and one deletion (Fig. 3). Blast searches against the Wheat Survey Sequence derived from the cultivar Chinese Spring indicated that *Pm2* has three homologues on chromosome 5DS sharing 98, 82 and 81% identity at the nucleotide level, respectively. The sequence of the closest homologue of *Pm2* on 5DS was almost identical to that of the susceptible *Pm2* allele (one SNP), suggesting that it is the same gene as the susceptible allele of *Pm2* described above. We also found two homologous sequences on chromosome 5BS (87 and 81% identity), and one on chromosome 5AS (95% identity). We also searched for *Pm2* homologous sequences in the barley High Confidence Genes database and found one sequence on chromosome 6H, MLOC_11605, with only 53% identity at the amino acid level (Table S9). The MLOC_11605 gene encodes for an NBS‐LRR protein annotated as a ‘Disease resistance protein’ that has 13 orthologues and three paralogues in barley (http://plants.ensembl.org). These data suggest that *Pm2* is a unique, single gene on wheat chromosome 5DS and there is no orthologue or closely related homologue in barley.

### *Pm2* induces a strong HR upon recognition of *AvrPm2*

**Figure 4:**
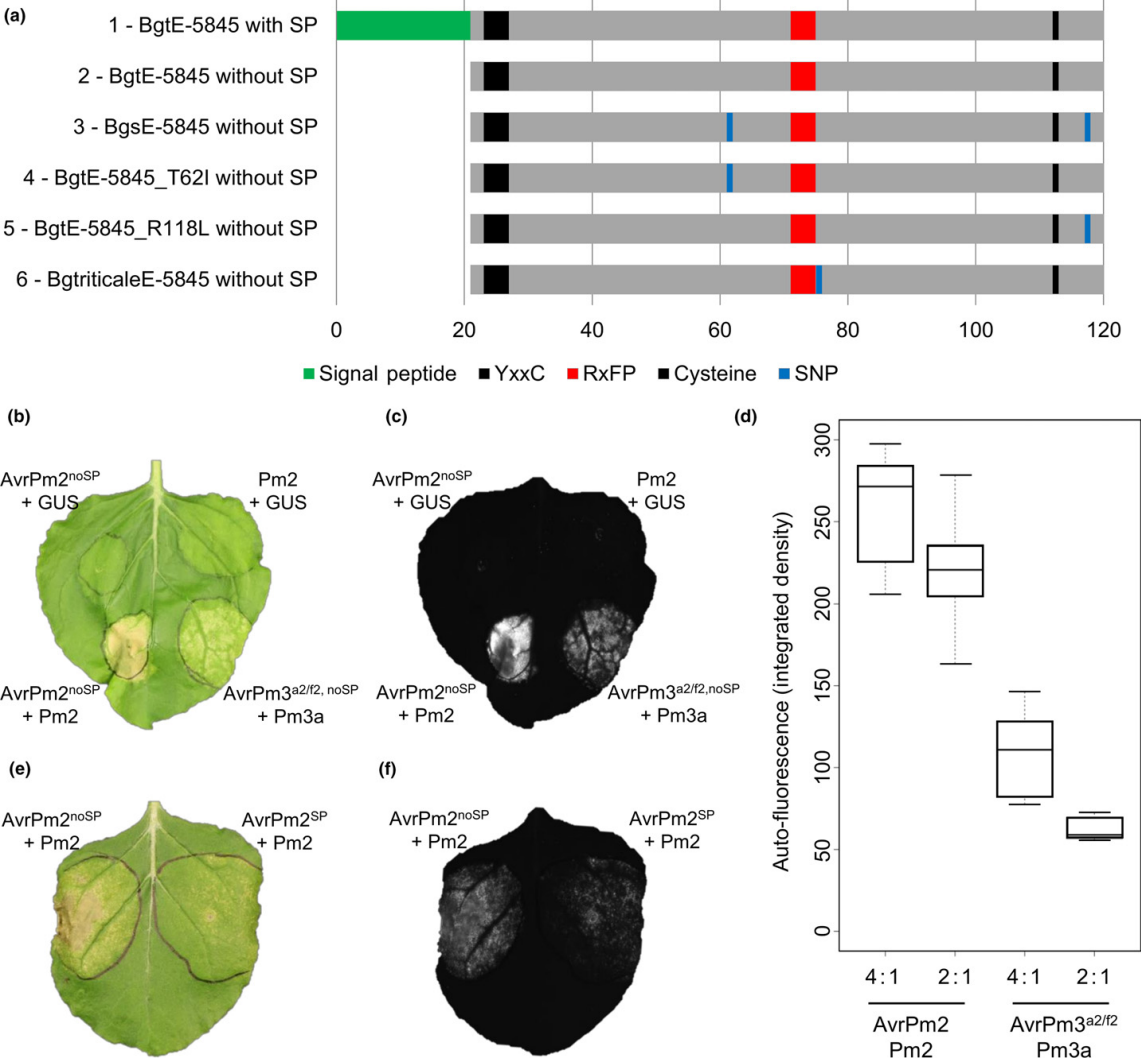
Functional validation of the *AvrPm2*–*Pm2* interaction by agroinfiltration in *Nicotiana benthamiana*. (a) Schematic diagram of the protein variants encoded by *AvrPm2*, its direct homologue in *Blumeria graminis* (*B. g*.) *secalis* and the variant identified in the *B. g. triticale* isolate CAP‐39‐A1 used for transient expression assays. ‘1’ represents the full‐length protein encoded by *AvrPm2* (120 residues) and ‘2’ the variant without signal peptide. The predicted signal peptide (SP, SignalP version4.1) is indicated in green. The locations of the Y(x)xC motif and the conserved cysteine in the C‐terminal region are indicated in black and the RxFP motif in red. Blue bars indicate single nucleotide polymorphisms (SNPs) distinguishing *BgtE‐5845* and the other variants. ‘3’ is the orthologue of *AvrPm2* in *B. g. secalis*, ‘4’ and ‘5’ are two variants containing only one of the two SNPs of *B. g. secalis*, and ‘6’ is the variant of the *B. g. triticale* isolate CAP‐39‐A1. (b, c) Agroinfiltration assays in *N. benthamiana* demonstrate induction of the hypersensitive response (HR) upon specific recognition of *AvrPm2* by *Pm2*. *AvrPm2* without signal peptide (AvrPm2^no^ ^SP^) was transiently expressed together with *Pm2* in a 4 : 1 Avr : R ratio. *AvrPm2* and *Pm2* were also co‐expressed with GUS as a control showing that *AvrPm2* and *Pm2* alone do not induce HR. As a positive control, *AvrPm3* ^*a2/f2*^ was co‐expressed with *Pm3a*. (d) Quantification and comparison of the HR induced upon co‐infiltration of *AvrPm2* and *Pm2* to that induced upon co‐infiltration of *AvrPm3* ^*a2/f2*^ and *Pm3a*, using two different Avr : R ratios (4 : 1 and 2 : 1). The fluorescence measurements are depicted in ‘integrated density’, which is the product of the area infiltrated and the mean grey value. (e, f) Agroinfiltration assays in *N. benthamiana* with *AvrPm2* constructs encoding the protein versions with and without signal peptide co‐infiltrated with *Pm2* (Avr : R ratio = 4 : 1). Stronger HR was observed with the *AvrPm2* construct expressing the protein without signal peptide. (c, f) Fluorescence imaging of the HRs obtained from assays in (b) and (e) used for HR quantification. For the assays in (b)–(f) leaves of 4‐wk‐old *N. benthamiana* plants were infiltrated with *Agrobacterium tumefaciens* cultures expressing each of the constructs indicated. Results were consistent across at least three independent replicates where at least four leaves were assayed. Photographs were taken 5 d after infiltration.

To functionally validate *AvrPm2*, we used the transient agroinfiltration assay in *N. benthamiana* described by Bourras *et al*. (2015). The *AvrPm2* gene was cloned from the avirulent parent 96224, and constructs expressing protein versions with and without signal peptide were transiently co‐expressed with *Pm2* in a 4 : 1 Avr : R ratio (optical density at 600 nm (OD_600_) = 1.2, Fig. 4a). We also co‐expressed the experimentally validated *AvrPm3* ^*a2/f2*^–*Pm3a Avr‐R* gene pair as a control (Fig. 4b,c). A strong hypersensitive cell death response was observed when *AvrPm2* was co‐expressed with *Pm2*, whereas no HR was observed when these constructs were co‐expressed with the GUS control (Fig. 4b,c). Visual inspection of the HR induced by *Pm2* in the presence of the native *AvrPm2* construct expressing the full‐length protein revealed a weaker HR compared to the *AvrPm2* construct without the signal peptide (Fig. 4e,f). These results demonstrate that AVRPM2 is the cognate avirulence protein of PM2, and suggest that the AVRPM2–PM2 interaction takes place in the cytosol or the nucleus. Similarly, visual inspection of several replicates of the *AvrPm2–Pm2* triggered HR indicated it was consistently stronger than the *AvrPm3* ^*a2/f2*^–*Pm3a* positive control. Therefore, we analysed the infiltrated *N. benthamiana* leaves by fluorescence imaging and quantified side by side the hypersensitive cell death response induced by *Pm2* and *Pm3* in the presence of 4 : 1 and 2 : 1 Avr : R ratios. We found that the HR induced by *Pm2* was always significantly stronger than *Pm3* independently of the Avr : R ratio (4 : 1, 2.3× stronger; 2 : 1, 3.5× stronger, Fig. 4d; Tables S10, S11). In addition, there was only a slight but not significant reduction in HR intensity when *Pm2* was co‐infiltrated with lower amounts of *AvrPm2* construct (2 : 1 ratio of Avr : R), whereas a significant HR reduction of 45% was observed for *Pm3* (Fig. 4d; Tables S10, S11). The HR was even weaker and not visible by eye for *Pm3* with a ratio of 2 : 1 while it was clear and strong for *Pm2* (Fig. S6).

**Figure 5:**
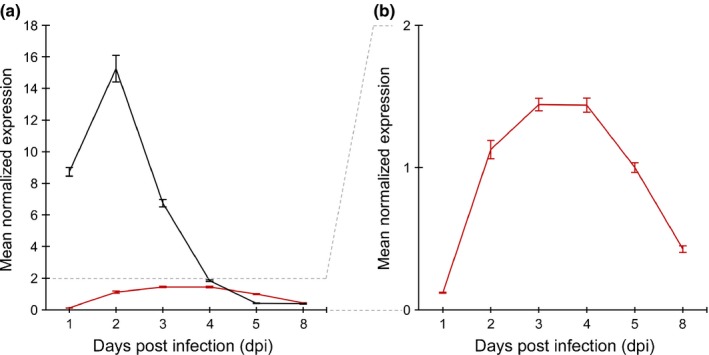
Gene expression of *AvrPm2* and *AvrPm3* ^*a2/f2*^ in the avirulent isolate 96224. (a) The mean normalized expression of *AvrPm2* (red line) and *AvrPm3* ^*a2/f2*^ (black line) in the avirulent parental isolate 96224, 1–8 d post infection (dpi) of the susceptible wheat genotype Chinese Spring. (b) Magnification of the lower mean gene expression of *AvrPm2* in isolate 96224, 1–8 dpi of Chinese Spring. Each data point is the average of three technical replicates. These results were consistent in three biological replicates as shown in Supporting Information Fig. S7. The error bars indicate the standard error of the mean (SEM).

We wanted to compare gene expression of *AvrPm2* and *AvrPm3* ^*a2/f2*^ in the avirulent parent 96224. We isolated RNA from leaf segments of the susceptible wheat cultivar Chinese Spring inoculated with the *Pm2* and *Pm3a* avirulent isolate 96224. Samples were collected at six time points from 1 to 8 d post‐inoculation (dpi), and relative gene expression over time was assessed by qRT‐PCR. *AvrPm2* is expressed at lower amounts than *AvrPm3* ^*a2/f2*^ and shows an almost constitutive expression over time (Figs 5a, S7). Scale magnification revealed very low mRNA amounts of *AvrPm2* at 1 dpi, and a mild but sustained increase in expression between 3 and 4 dpi that is evidence for a shift of gene expression kinetics towards later infection stages as compared to *AvrPm3* ^*a2/f2*^ (Fig. 5b).

### AVRPM2 belongs to a conserved family of RNase‐like effectors

**Figure 6:**
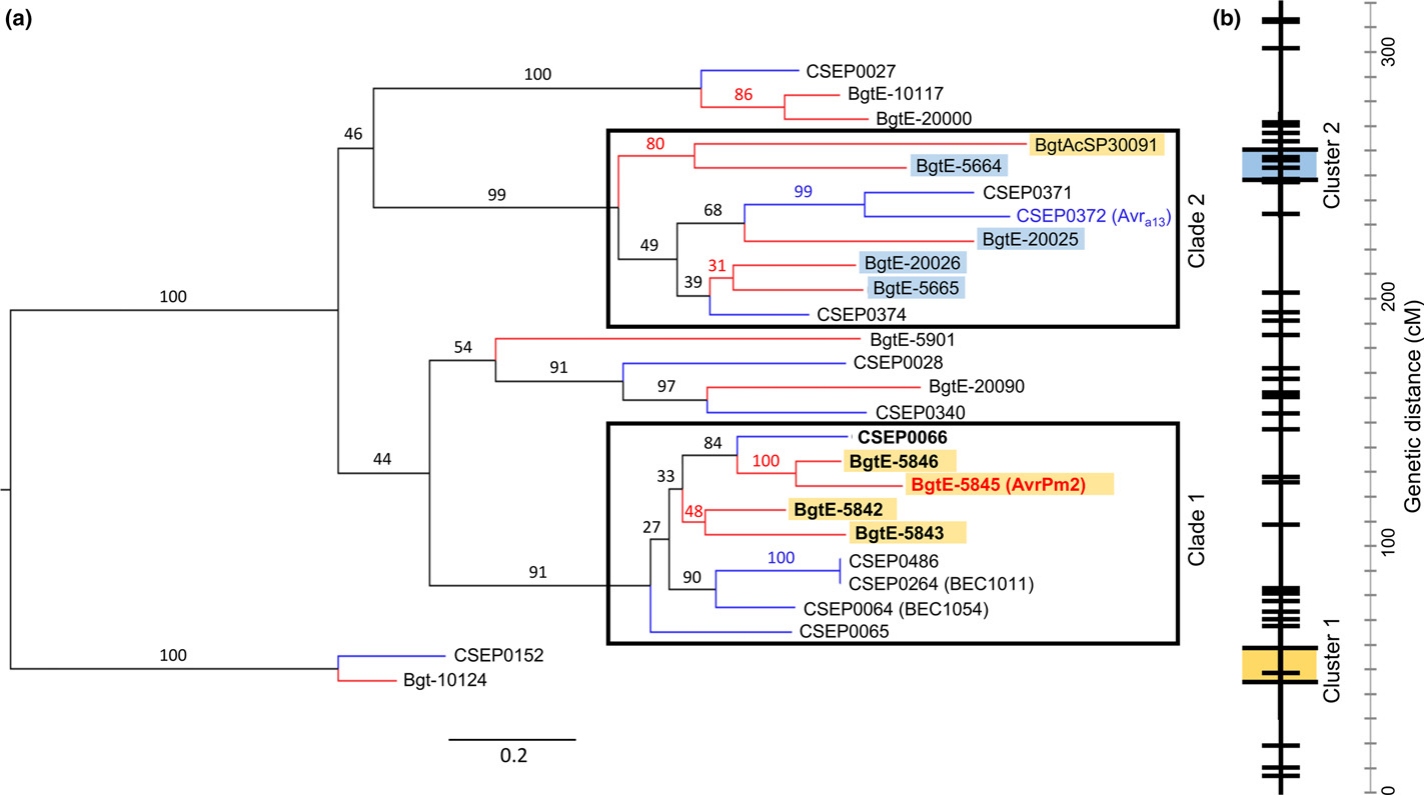
Phylogenetic analysis of the *AvrPm2* family. (a) Maximum likelihood tree of the *AvrPm2* family in *Blumeria graminis* (*B. g. *) *tritici* and *B. g. hordei*. The tree was inferred with the complete nucleotide sequences. Red branches, *B. g. tritici* genes; blue branches, *B. g. hordei* genes. The two framed boxes indicate clade 1 and clade 2. The genes highlighted in yellow are part of cluster 1 in the genetic map and those highlighted in blue are part of cluster 2. *AvrPm2* (*BgtE‐5845*) is indicated in red, and *Avr*_*a13*_ (*CSEP0372*) in blue. The alternative names of the *B. g. hordei* genes *CSEP0064* and *CSEP0264* are indicated in parentheses. The genes tested for recognition by *Pm2* by transient assays in *Nicotiana benthamiana* are indicated in bold. The scale bar indicates a measure of expected substitutions per site. Bootstrap values are indicated on the branches. (b) Consensus linkage group (Bourras *et al*., 2015) with clusters 1 and 2 indicated in yellow and blue, respectively. *AvrPm2* (*BgtE‐5845*) is located in cluster 1. The scale bar indicates the genetic distance (cM).

We found an effector family of *AvrPm2* homologues that contained 24 members, 14 from *B. g. tritici* and ten from *B. g. hordei*. In *B. g. tritici*, the family includes all four predicted effector genes encoded within the *AvrPm2* locus (Fig. 1d; Methods S4), and an additional effector, *BgtAcSP‐30091*, located on contig 48 that is genetically linked to contig 51 and these are hereafter referred to as ‘Cluster_1’ (Fig. 6b). We found a second genetically unlinked and physically distant cluster of four members (Cluster_2) located on the same linkage group as *AvrPm2* (Fig. 6b). Five additional members were found as singletons in genetically unlinked sequences in contigs 33, 62 and 133 or in unassembled sequences for which we have no genetic data. The Y(x)xC motif was conserved among the gene family, and an additional conserved protein motif consisting of one arginine, followed by a non‐conserved residue, a phenylalanine or a tyrosine, and a highly conserved proline that we refer to as the ‘RxFP motif’ (Fig. S8). A disulphide bridge between the conserved cysteine residues was predicted in nine out of the 14 *B. g. tritici* family members including *AvrPm2* (Table S12). Phylogenetic analyses were performed using the sequences encoding the full protein as well as the mature peptide after removal of the secretion signal and including all family members from both wheat and barley mildews. The phylogenetic trees were identical independently from the presence/absence of the signal peptide and revealed several gene duplication and gene loss events after the divergence between those two *formae speciales* of mildew. We then tested for positive selection as described in Yang *et al*. (2000, 2005) and found that the *AvrPm2* family is under diversifying selection, with the sequence after the signal peptide region accumulating positively selected amino acids (Fig. S8). These results are similar to those reported for *AvrPm3* ^*a2/f2*^, suggesting common patterns of evolution among *Avr* effector families in wheat powdery mildew.

**Figure 7:**
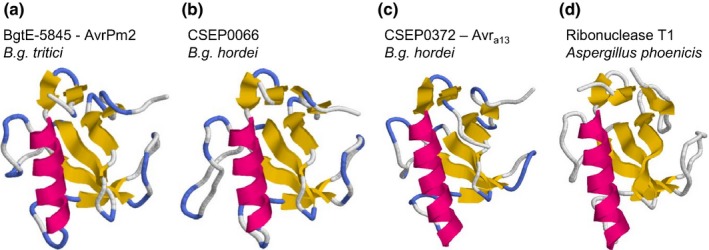
Three‐dimensional protein models of AVRPM2, its closest homologue in *Blumeria graminis* (*B. g*.) *hordei*, and AVR_a13_. Three‐dimensional protein models for (a) BgtE‐5845 (AVRPM2), (b) the closest AVRPM2 homologue in *B. g. hordei* CSEP0066 and (c) AVR_a13_ (synonym: CSEP0372). (d) The known crystal structure of ribonuclease T1 from *Aspergillus phoenicis*, which was found to be the best template for the AVRPM2 family by the raptorX structure prediction server (http://raptorx.uchicago.edu/).

One member of the *AvrPm2* family in *B. g. hordei*, CSEP0372, is *Avr*_*a13*_ that is recognized by the barley powdery mildew resistance gene *Mla13* (Lu *et al*., 2016). Two additional members of the *AvrPm2* homologous family in *B. g. hordei* are the well‐characterized mildew CSEPs BEC1011 and BEC1054 (Pedersen *et al*., 2012; Pliego *et al*., 2013; Pennington *et al*., 2016). Previous studies have shown that BEC1011 and BEC1054 belong to the RNase‐like class of mildew effectors (Pedersen *et al*., 2012). Therefore, we tested all the members of the *AvrPm2* family from wheat and barley powdery mildew in structural protein modelling predictions using RaptorX (Källberg *et al*., 2012). Consistent with the previously predicted RNase‐like structure of BEC1011 and BEC1054, we found structural homologies to fungal ribonucleases throughout the *AvrPm2* family in both wheat and barley mildews (Fig. 7; Table S13). This demonstrates that two members of the RNase‐like class of effectors act as avirulence factors in powdery mildews.

### *AvrPm2* is a conserved and functional *Avr* in rye and triticale powdery mildews

We also searched the rye and triticale mildew genomes for *AvrPm2* orthologous genes. In rye mildew (*B. g. secalis*), we found an orthologue, *BgsE‐5845*, that differs from *BgtE‐5845* by two amino acids (Fig. 4a). We found no polymorphism in the *BgsE‐5845* sequence among the five *B. g. secalis* isolates we analysed (Menardo *et al*., 2016). In triticale mildew (*B. g. triticale*), which is genetically very similar to wheat mildew, we identified only one variant of *AvrPm2* carrying a single mutation in the isolate CAP‐39‐A1 (*BgtriticaleE‐5845*) (Fig. 4a). By contrast, we found no direct homologue to *AvrPm2* in the barley powdery mildew genome, and the closest gene was *CSEP0066* (48% identity at the amino acid level).

All *AvrPm2* haplotypes and direct/closest homologues identified from triticale, rye and barley powdery mildews were tested for recognition by *Pm2* using transient assays in *N. benthamiana*. We also tested *BgtE‐5842*,* BgtE‐5843* and *BgtE‐5846*, three additional effectors encoded within the *AvrPm2* locus (Fig. 1c). All transient co‐expression assays involving the additional effectors encoded within the *AvrPm2* locus and the closest *AvrPm2* homologue in *B. g. hordei* resulted in no HR, indicating these effectors are not recognized by *Pm2* (Fig. S6). However, all transient co‐expression assays involving the *AvrPm2* haplotype in *B. g. triticale* (*BgtriticaleE‐5845*), the *B. g. secalis* homologue (*BgsE‐5845*) as well as two *AvrPm2* variants harbouring either one of the SNPs identified in *BgsE‐5845* (*BgtE‐5845_T62I* and *BgtE‐5845_R118C*) resulted in strong HR, indicating conservation of a functional *AvrPm2* gene in powdery mildew forms specialized on wheat, triticale and rye (Figs 4a, S6).

## Discussion

In this study we cloned the mildew avirulence gene *AvrPm2* and showed that it is not related to the only previously known avirulence gene in *B. g. tritici*,* AvrPm3* ^*a2/f2*^. *AvrPm2* is a member of the family of RNAse‐like effectors that includes *Avr*_*a13*_, a barley mildew effector recognized by the *Mla13* resistance gene. We also showed that *Pm2* recognizes a homologue of *AvrPm2* from rye powdery mildew, *BgsE‐5845*.

### Cloning and functional validation of avirulence genes in powdery mildews

For this work, we combined map‐based cloning, next generation sequencing and high‐throughput genotyping to clone *AvrPm2* in a similar approach to that used for the identification of *AvrPm3* ^*a2/f2*^ (Bourras *et al*., 2015). Given that map‐based cloning is time consuming, we also used GWAS to identify *AvrPm2* from a panel of 60 natural isolates showing a balanced pattern of virulence on *Pm2* (33 avirulent, 27 virulent). We used whole genome sequencing data for GWAS and the best associated SNP for *AvrPm2* correctly identified the genetic region, but was actually below the threshold for statistical significance after standard correction for multiple testing. It was located in a non‐genic region at 50 kb from a large 12 kb deletion that contained the *Avr*. Thus, the use of genomic sequences instead of transcriptomics was crucial for finding *AvrPm2* because the SNP was located in a non‐genic region. In barley powdery mildew, an approach using transcriptomic data led to the cloning of two novel avirulence genes, *Avr*_*a1*_ and *Avr*_*a13*_ (Lu *et al*., 2016). The two case studies now available demonstrate that association studies in a geographically diverse set of isolates can accelerate *Avr* gene cloning in powdery mildews.

We found by transient expression in *N. benthamiana* that *Pm2* recognizes *AvrPm2* and that the HR observed in the *AvrPm2–Pm2* interaction is stronger than that in the *AvrPm3* ^*a2f2*^ *–Pm3a* interaction. In addition, *Pm2* can recognize smaller ratios of *Avr* constructs. Quantification was based on fluorescence imaging, which specifically allows the visualization of the accumulation of phenolic compounds associated with the HR in *N. benthamiana* (Chaerle & Van Der Straeten, 2000; Chaerle *et al*., 2007). This method also allows rapid inspection of the area undergoing HR and the detection of weak responses that are not visible or difficult to reveal by classical chemical or histochemical methods (e.g. Trypan blue staining; Ma *et al*., 2012). Fluorescence can be precisely quantified using standard image analysis software such as ImageJ (Schindelin *et al*., 2015) and the data can then be normalized and differences can be statistically tested. In addition, no histochemical pre‐treatment or staining is required, which makes it very suitable for screening large numbers of interactions.

### The genetic and molecular basis of avirulence in powdery mildews

On the pathogen side, avirulence to *Pm2* is controlled by an effector encoding gene whose virulent allele is the result of a 12 kb deletion that includes the *Avr* gene. In the case of the *AvrPm2* deletion, the genomic context might have played a role: the low‐copy region containing *AvrPm2* is flanked by five *Copia* retrotransposons that all belong to the *Ino* family, and which all share at least 80% sequence identity. Most importantly, the two elements flanking the *AvrPm2* region are in the same transcriptional orientation. Thus, it is possible that this sequence organization provided the template for an unequal recombination event that led to the deletion of the low‐copy region containing *AvrPm2* (Fig. S9; Georgiev *et al*., 1989). Additional examples of gain of virulence by partial or complete deletion of the *Avr* gene have been described in barley powdery mildew (e.g. *Avr*_*a13*_; Lu *et al*., 2016) and the hemibiotroph pathogen *Magnaporthe oryzae* (e.g. *AvrPib*; Zhang *et al*., 2015), indicating that gene loss is a common mechanism for *Avr* turnover in biotrophic fungi. This is very different from the *AvrPm3* ^*a2/f2*^ *–Pm3a/f* interaction, where gain of virulence is mediated either by point mutations of the AVR protein or the action of a suppressor of *Avr* recognition (Bourras *et al*., 2015). In flax rust, another obligate biotrophic fungus, gain of virulence is exclusively based on amino acid polymorphism for all AVRs cloned so far. There, all *Avr–R* pairs can be described as gene‐for‐gene interactions, although an additional gene whose identity and mode of action are still unknown can act as a suppressor of *Avr* recognition (Ellis *et al*., 2007; Ravensdale *et al*., 2011). Thus, there are additional layers of complexity and a larger variety of genetic mechanisms controlling *Avr–R* interactions in powdery mildews that have not yet been identified in other fungal systems. These can range from a simple gene‐for‐gene model (*AvrPm2*–*Pm2*;* Avr*_*a13*_–*Mla13*) to epistatic interactions and multi‐loci determinism that is reminiscent of a quantitative trait (*AvrPm3*–*Pm3*).

### Structure and evolution of the AVRPM2 effector family

*AvrPm2* belongs to an effector family of 26 members that is sequence unrelated and structurally distinct from the *AVRPM3* ^*A2/F2*^ family. In addition to a conserved Y(x)xC‐like motif found at the N‐terminal end of the predicted mature peptide, the *AVRPM2* family also shares an RxFP motif separating the two N‐ and C‐terminal highly variable regions, which is reminiscent of the RXLR–dEER motif in oomycetes (Dou *et al*., 2008). There, it was shown that these motifs are sufficient for the translocation of pathogen effectors into the host cytoplasm (Dou *et al*., 2008). Therefore, the strong conservation of the Y(x)xC–RxFP motifs in otherwise highly divergent effector sequences suggests an important role of these residues in effector protein function.

The *AvrPm2* family encodes effectors with structural homology to ribonucleases (RNase‐like) and contains the barley *Avr*_*a13*_ gene as well as BEC1011 and BEC1054. The latter two effectors have been shown to be important for mildew virulence (Pliego *et al*., 2013; Pennington *et al*., 2016). *AvrPm2* and *Avr*_*a13*_ are homologues and encode structurally conserved effectors. However, their cognate resistance genes, *Pm2* and *Mla13*, encode sequence‐unrelated and evolutionary distinct NLRs from wheat and barley. Assuming direct AVR–R binding in these interactions, this could be explained by convergent evolution of PM2 and MLA13, resulting in recognition of structurally conserved effectors. Another possible hypothesis is that AVRPM2 and AVR_a13_ both interact with a conserved protein in wheat and barley that is guarded by PM2 and MLA13, respectively. The AVRPM2–PM2 and the AVR_a13_–MLA13 interactions can be functionally studied by site‐directed mutagenesis and domain swaps of the two AVR proteins. Such studies, in addition to biochemical work, will result in a deeper understanding of recognition specificity and reveal whether there is convergent evolution of effector recognition or conservation of Avr targets.

### Conservation of *Avr* function in powdery mildew forms specialized on different hosts

We found that all *B. g. tritici* avirulent isolates contained the same haplotype of *AvrPm2*. Two additional haplotypes were found in the *B. g. triticale* isolate CAP‐39‐A1 and in the orthologous gene of *AvrPm2* in *B. g. secalis*, indicating conservation of *AvrPm2* in wheat, triticale and rye powdery mildews. *B. g. triticale* is a hybrid of *B. g. tritici* and *B. g. secalis*, two mildew *formae speciales* that diverged *c*. 200 000 yr ago which is quite recent compared to their hosts, wheat and rye that diverged 4 million yr ago (Middleton *et al*., 2014). In all *Pm2* virulent isolates, we found a very similar 12 kb deletion, independent from the geographical origin (Europe, Israel, China), indicating that the virulent *avrPm2* allele is also conserved, and suggesting there is no naturally occurring virulent protein variant for AVRPM2. A contrasting example can be found in *Magnaporthe oryzae* where the virulent alleles of *AvrPib* arose from a variety of gain‐of‐virulence mechanisms including TE insertion, segmental deletion, complete gene deletion and point mutations (Zhang *et al*., 2015). There are different possible explanations for the presence of very similar or identical deletions in isolates from different geographical origins: either the *AvrPm2* deletion is ancient and occurred before the global cultivation of wheat and rye, or the deletion is associated with a better fitness of the pathogen and spread rapidly around the world after its occurrence.

Many disease resistance genes in bread wheat have been introgressed from wild and cultivated related species. These include genes such as *Pm21* introgressed from the wild species *Haynaldia villosa* (Cao *et al*., 2011) or the race‐specific *Pm8* and *Pm17* mildew resistance genes from rye (Yahiaoui *et al*., 2006; Hurni *et al*., 2013). It has long been hypothesized that the successful transfer of functional *R* genes from one species to another is based on the recognition of effectors conserved in pathogens specialized on different host species. *AvrPm2* is highly conserved in wheat, triticale and rye mildews, and all variants in these *formae speciales* can act as the cognate *Avr* of *Pm2*. Therefore, *AvrPm2* provides molecular evidence that functional *R* gene introgressions could indeed be based on recognition of effectors conserved in powdery mildew forms specialized on specific hosts. The identification of such conserved *Avrs* will help to detect redundant specificities of resistance genes in the gene pool used for wheat breeding. Such knowledge can be used in pre‐breeding to develop pathogen‐informed approaches for the identification of mildew resistance genes with non‐redundant effector recognition in wild and domesticated relatives that can then be introgressed into cereal crops. 

## Author contributions

C.R.P., S.B., D.Y. and B.K. designed the study. C.R.P., S.B., F.Z., J.S‐M., R.B., G.H., K.E.M., R.B‐D., F.P. and S.F. performed the experiments. C.R.P., S.B., F.Z., D.Y. and B.K. wrote and edited the manuscript. C.R.P., F.M., S.R., S.O., L.K.S. and T.W. performed analysis. F.Z., M.X., L.Y. and R.B.D. contributed data.

## Supporting information

Please note: Wiley Blackwell are not responsible for the content or functionality of any Supporting Information supplied by the authors. Any queries (other than missing material) should be directed to the *New Phytologist* Central Office.

**Fig. S1** Primer efficiency curve for qRT‐PCR primers for *AvrPm2*.
**Fig. S2** Genetic and physical mapping based on the *B. g. triticale/B. g. hordei* genome collinearity.
**Fig. S3** Assembly of the BACs spanning the *AvrPm2* region.
**Fig. S4** Identification of the *AvrPm2* deletion in the *Pm2* virulent isolate JIW2.
**Fig. S5** Gel electrophoresis analysis of *AvrPm2* deletion markers.
**Fig. S6** Functional analysis of the interaction between *Pm2* and different family members and homologues in *B. g. triticale*,* B. g. secalis* and *B. g. hordei* in *N. benthamiana*.
**Fig. S7** Gene expression of *AvrPm2* and *AvrPm3* ^*a2/f2*^ in the avirulent isolate 96224.
**Fig. S8** Annotated protein alignment of the AVRPM2 effector family in *B. g. tritici* and *B. g. hordei*.
**Fig. S9** Proposed molecular mechanism that could lead to the loss of the genomic region containing the *AvrPm2* gene.
**Table S1** Summary of the RNA samples used for qRT‐PCR
**Table S2** List of markers used for gene annotation, cloning and expression analysis
**Table S3** List of markers used for genetic mapping
**Table S4** BAC end markers
**Table S5** Markers used for estimating the size of the deletion
**Table S6** Whole genome resequencing strategies for the 22 Chinese isolates
**Table S9** Blast hits of PM2 against wheat and barley databases
**Table S10** Results of the ANOVA for HR intensity in HR quantification assays
**Table S11** Results of Tukey HSD *post‐hoc* test
**Table S12** Signal peptide and disulphide bond predictions in the AVRPM2 family
**Table S13** Structure prediction of the AVRPM2 in *B. g. tritici* and *B. g. hordei*
**Methods S1** BAC selection and sequencing.
**Methods S2** Illumina whole‐genome resequencing.
**Methods S3** RNA extraction and qRT‐PCR assays.
**Methods S4** Genome‐wide effector annotation and *AvrPm2* family identification in different *formae speciales*.
**Methods S5** *In silico* analysis of proteins encoded by *Pm2* and *AvrPm2*.

**Table S7** List of the *B. g. tritici* and *B. g. triticale* isolates used for genome‐wide association studies for the identification of *AvrPm2*

**Table S8** Genetic map of the 96224 × JIW2 mapping population based on KASP markers

**Notes S1** Sequences of the *Blumeria graminis* genes used for transient expression in *Nicotiana benthamiana*.

**Notes S2** Coding sequences of the *AvrPm2* family members in *B. g. tritici* and *B. g. hordei*.

**Notes S3** Protein sequences encoded by *AvrPm2* family members in *B. g. tritici* and *B. g. hordei*.
**Notes S4** BAC selection, sequencing and assembly.
**Notes S5** Genetic and physical mapping of *AvrPm2* based on the *B. g. tritici*/*B. g. hordei* genome collinearity.
