# Supplementary material for: AvrPm2 encodes an RNase‐like avirulence effector which is conserved in the two different specialized forms of wheat and rye powdery mildew fungus
Source: New Phytol. 2016 Dec 9;213(3):1301–14. doi: 10.1111/nph.14372 (PMC5347869; doi:10.1111/nph.14372)
Supplement: Supplementary file 1 — Fig. S1 Primer efficiency curve for qRT‐PCR primers for AvrPm2. Fig. S2 Genetic and physical mapping based on the B. g. triticale/B. g. hordei genome collinearity. Fig. S3 Assembly of the BACs spanning the AvrPm2 region. Fig. S4 Identification of the AvrPm2 deletion in the Pm2 virulent isolate JIW2. Fig. S5 Gel electrophoresis analysis of AvrPm2 deletion markers. Fig. S6 Functional analysis of the interaction between Pm2 and different family members and homologues in B. g. triticale, B. g. secalis and B. g. hordei in N. benthamiana. Fig. S7 Gene expression of AvrPm2 and AvrPm3 a2/f2 in the avirulent isolate 96224. Fig. S8 Annotated protein alignment of the AVRPM2 effector family in B. g. tritici and B. g. hordei. Fig. S9 Proposed molecular mechanism that could lead to the loss of the genomic region containing the AvrPm2 gene. Table S1 Summary of the RNA samples used for qRT‐PCR Table S2 List of markers used for gene annotation, cloning and expression analysis Table S3 List of markers used for genetic mapping Table S4 BAC end markers Table S5 Markers used for estimating the size of the deletion Table S6 Whole genome resequencing strategies for the 22 Chinese isolates Table S9 Blast hits of PM2 against wheat and barley databases Table S10 Results of the ANOVA for HR intensity in HR quantification assays Table S11 Results of Tukey HSD post‐hoc test Table S12 Signal peptide and disulphide bond predictions in the AVRPM2 family Table S13 Structure prediction of the AVRPM2 in B. g. tritici and B. g. hordei Methods S1 BAC selection and sequencing. Methods S2 Illumina whole‐genome resequencing. Methods S3 RNA extraction and qRT‐PCR assays. Methods S4 Genome‐wide effector annotation and AvrPm2 family identification in different formae speciales. Methods S5 In silico analysis of proteins encoded by Pm2 and AvrPm2. [file NPH-213-1301-s001.pdf]

New Phytologist

***New Phytologist* Supporting Information**

Article title: ***AvrPm2* encodes an RNase-like avirulence effector which is conserved in the two different specialized forms of wheat and rye powdery mildew fungus**

Authors: Coraline R. Praz, Salim Bourras, Fansong Zeng, Javier Sánchez-Martín, Fabrizio Menardo, Minfeng Xue, Lijun Yang, Stefan Roffler, Rainer Böni, Gerard Herren, Kaitlin E. McNally, Roi Ben-David, Francis Parlange, Simone Oberhaensli, Simon Flückiger, Luisa K. Schäfer, Thomas Wicker, Dazhao Yu and Beat Keller

Article acceptance date: 2 November 2016

The following Supporting Information is available for this article:

Fig. S1 Primer efficiency curves for qRT-PCR primers for *AvrPm2.*

Fig. S2 Genetic and physical mapping based on the *B.g. tritici*/*B.g. hordei* genome collinearity.

Fig. S3 Assembly of the BACs spanning the *AvrPm2* region.

Fig. S4 Identification of the *AvrPm2* deletion in the *Pm2* virulent isolate JIW2.

Fig. S5 Gel electrophoresis analysis of *AvrPm2* deletion markers.

Fig. S6 Functional analysis of the interaction between *Pm2* and different family members and homologs in *B.g. triticale, B.g. secalis* and *B.g. hordei* in *N. benthamiana.*

Fig. S7 Gene expression of *AvrPm2* and *AvrPm3*$^{a2/f2}$ in the avirulent isolate 96224.

Fig. S8 Annotated protein alignment of the AVRPM2 effector family in *B.g. tritici* and *B.g. hordei.*

Fig. S9 Proposed molecular mechanism that could lead to the loss of the genomic region containing the *AvrPm2* gene.

Table S1 Summary of the RNA samples used for qRT-PCR.

Table S2 List of markers used for gene annotation, cloning and expression analysis.

Table S3 List of markers used for genetic mapping.

Table S4 BAC end markers.

Table S5 Markers used for estimating the size of the deletion.

Table S6 Whole genome resequencing strategies for the 22 Chinese isolates.

Table S7 List of the *B.g. tritici* and *B.g. triticale* isolates used for genome-wide association studies for the identification of *AvrPm2*. (See separate file.)

Table S8 Genetic map of 96224 x JIW2 mapping population based on KASP markers. (See separate file.)

Table S9 BLAST hits of PM2 against wheat and barley databases.

Table S10 Results of the ANOVA for HR intensity in HR quantification assays.

Table S11 Results of TukeyHSD post-hoc test.

Table S12 Signal peptide and disulfide bond predictions in the AVRPM2 family.

Table S13 Structure prediction of the AVRPM2 in *B.g. tritici* and *B.g. hordei.*

Methods S1 BAC selection and sequencing.

Method S2 Illumina whole-genome resequencing.

Methods S3 RNA extraction and qRT-PCR assays

Methods S4 Genome-wide effector annotation and AvrPm2 family identification in different formae speciales.

Methods S5 *In silico* analysis of proteins encoded by *Pm2* and *AvrPm2*

Notes S1 Sequences of the *Blumeria graminis* genes used for transient expression in *Nicotiana benthamiana.* (See separate file.)

Notes S2 CDS sequences of the *AvrPm2* family members in *B.g. tritici* and *B.g. hordei*. (See separate file.)

Notes S3 Protein sequences of the *AvrPm2* family members in *B.g. tritici* and *B.g. hordei*. (See separate file.)

Notes S4 BAC selection, sequencing and assembly.

Notes S5 Genetic and physical mapping of *AvrPm2* based on the *B.g. tritici*/*B.g. hordei* genome collinearity.

Fig. S1 Primer efficiency curves for qRT-PCR primers for *AvrPm2*. Primer efficiency was performed with dilution series (1x, 4x, 16x, 64x) and efficiency was calculated with Bio-Rad Real-Time PCR CFX Manager Software version 3.5

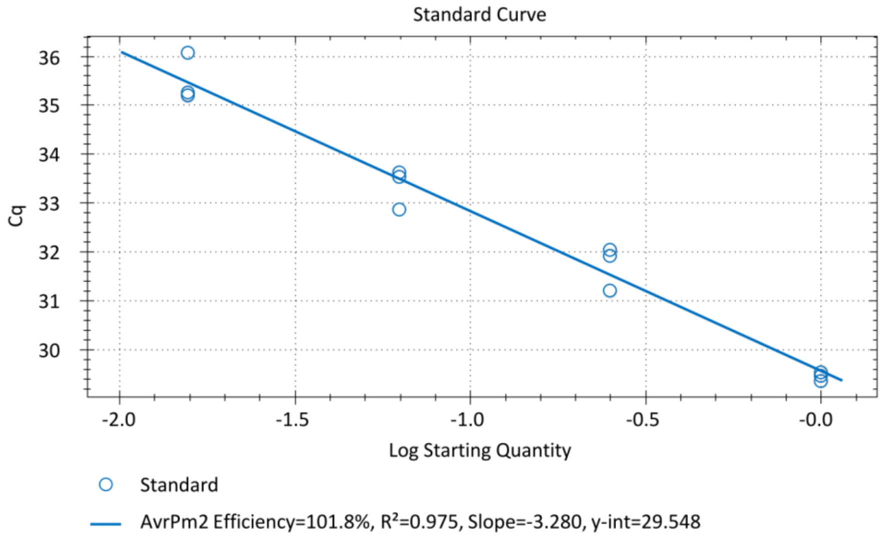

Fig. S2 Genetic and physical mapping based on the *B.g. tritici*/*B.g. hordei* genome collinearity. (a) Contig 47 of the *B.g. hordei* genome identified by BLAST searches using genes on contig 51 of *B.g. tritici*. Colored arrowheads represent genes. The direction of the arrowheads represents the transcriptional orientation of the gene. (b) Schematic representation of the *AvrPm2* locus based on *B.g. tritici*/*B.g. hordei* genome collinearity. Contigs 51 and 48 from *B.g. tritici* are shown. Colored arrowheads represent genes. The positions of the genes *BgtE-5842* and *Bgt-2686* were validated by genetic mapping. The direction of the arrowheads represents the transcriptional orientation of the gene. (c) Original arrangement of the *AvrPm2* locus on contig 51 in the *B.g. tritici* genome based on the FPC assembly. (d) Original contig 51 assembly in the *B.g. tritici* reference genome. For *B.g. tritici* contig 51 in (b–d), the regions with available genomic sequences are represented in dark grey and the gaps in light grey (Wicker *et al.*, 2013).

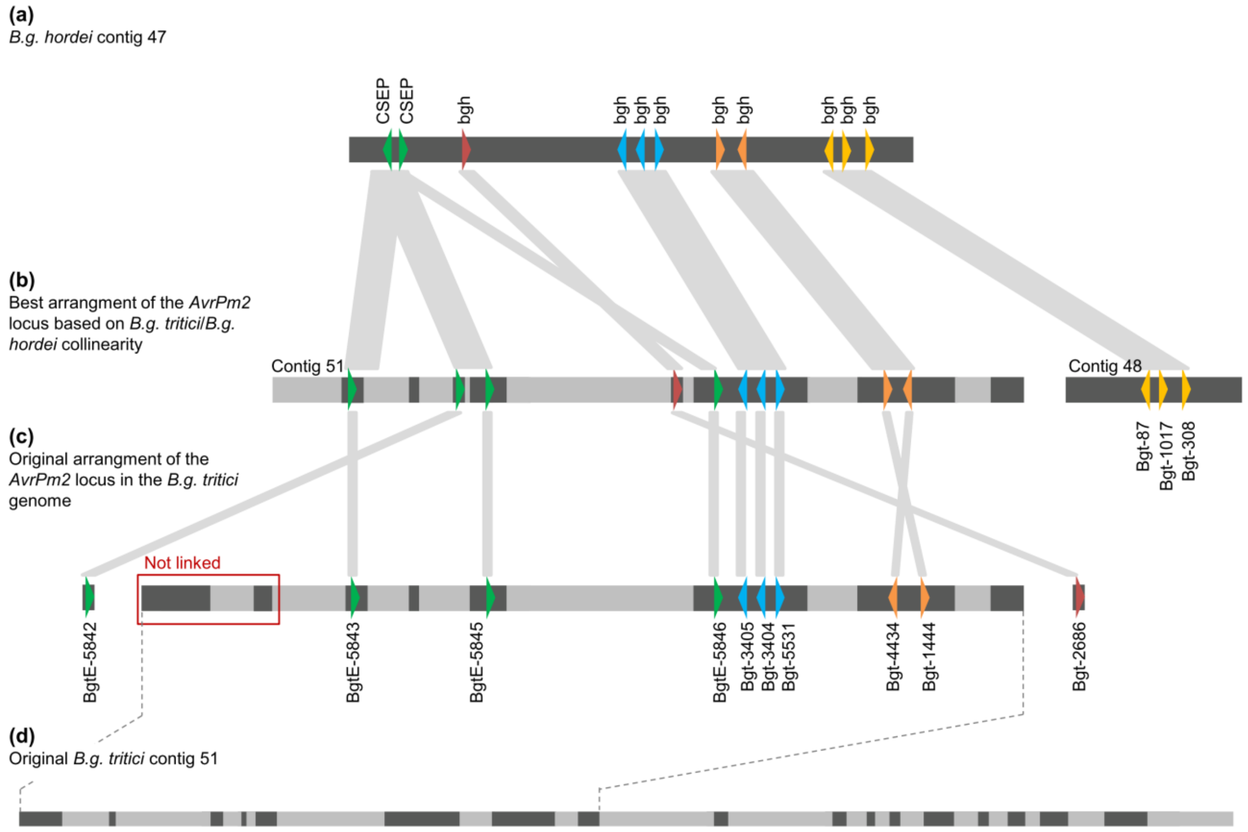

Fig. S3 Assembly of the BACs spanning the *AvrPm2* region. (a) Minimum tiling path of the FPC (left) and LTC (right) assemblies with informative genetic markers in colors and the informative BAC end markers in grey. (b) Individual sequence assemblies of the five selected BACs. Plain horizontal bars represent the contigs obtained using permissive assembly parameters while the striped bars represent the contigs obtained with stringent assembly parameters (Supporting Information Methods S1 and Notes S4). The contigs or contig segments used for the final assembly of the *AvrPm2* locus are indicated with a black asterisk. (c) Schematic representation of the model assembly of the *AvrPm2* region created from four BACs. The positions of the flanking markers are indicated and the four annotated genes are indicated with arrowheads. The *AvrPm2* physical interval is indicated with an orange double-headed arrow.

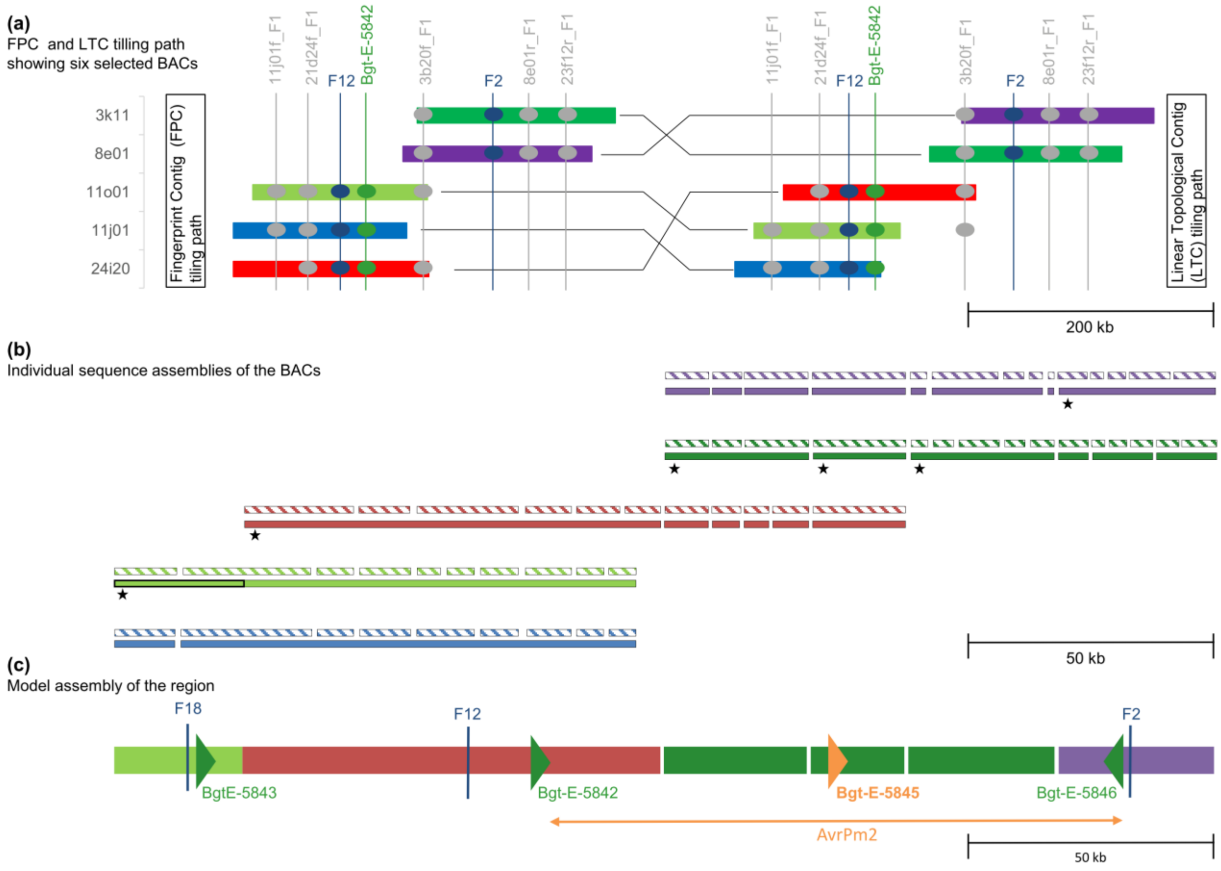

Fig. S4 Identification of the *AvrPm2* deletion in the *Pm2* virulent isolate JIW2. (a) Visual representation of the mapping of genome sequencing reads from the virulent parent JIW2 on the *AvrPm2* locus assembly. About 100 kb around *AvrPm2* (*BgtE-5845*) are represented. The position of *BgtE-5845* is indicated with an orange vertical line. No reads are mapping in and around *BgtE-5845* indicating a sequence deletion in the virulent parent JIW2. (b) Annotation of 40 kb around *AvrPm2* in the mildew isolate 96224. The gene *BgtE-5845* is indicated with an orange box. The other boxes indicate transposable elements: Copia LTR retrotransposons are indicated in red, Gypsy LTR retrotransposons in yellow and other LTR retrotransposons in grey. The deleted sequence in JIW2 as identified by coverage analysis is indicated with a black frame (Fig 2). (c) PCR validation of the *AvrPm2* deletion in three isolates. Primers were designed every 500 bp and spanning 50 kb were tested in the two parental isolates 96224 (avirulent), JIW2 (virulent) and in a third isolate (94202, avirulent). Black lines show the region where amplifications were obtained in the respective isolates. In the virulent isolate JIW2, a region of about 12 kb (508–520 kb) is deleted. A few unspecific amplifications were obtained inside the deleted segment from sequences corresponding to transposable elements.

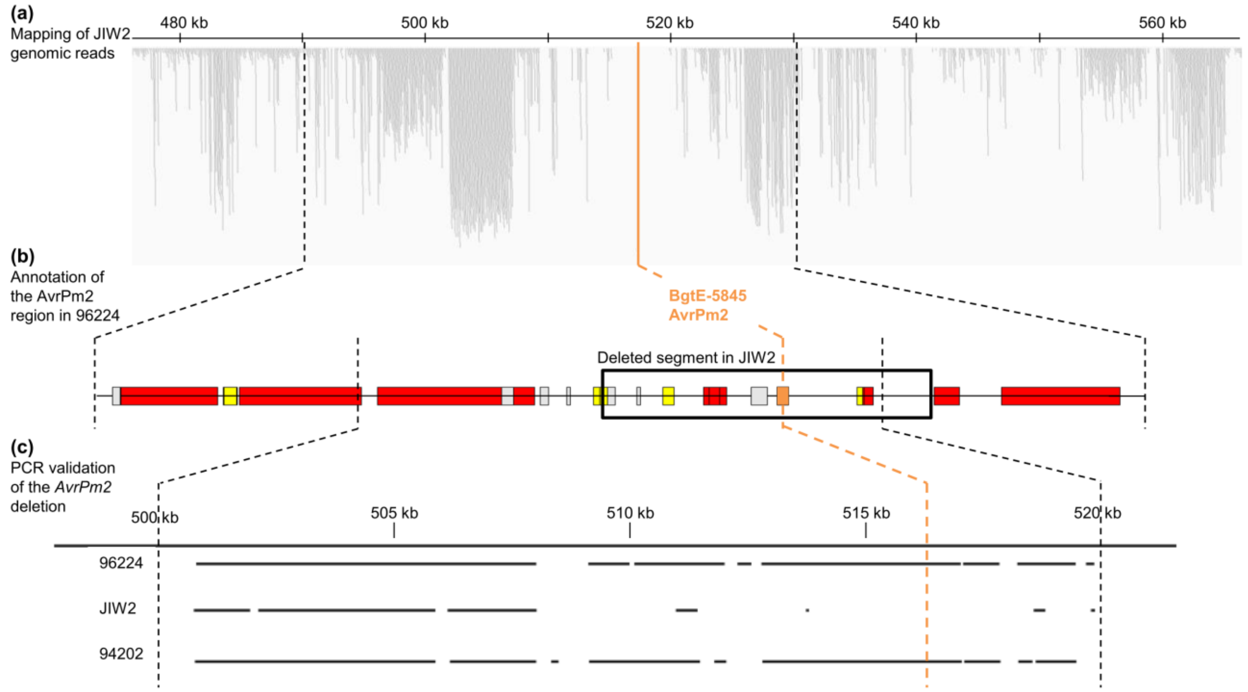

Fig. S5 Gel electrophoresis analysis of *AvrPm2* deletion markers. PCR amplifications of deletion markers 1 to 21 in the isolates 96224, JIW2 and 94202 are depicted. Fragment size length is indicated with a DNA ladder. Markers 1–15 illustrate even amplification in all isolates. Markers 16 and 17 illustrate nonfunctional markers. Markers 18–21 illustrate unspecific or no amplification in JIW2. All fragments were subsequently verified by sequencing.

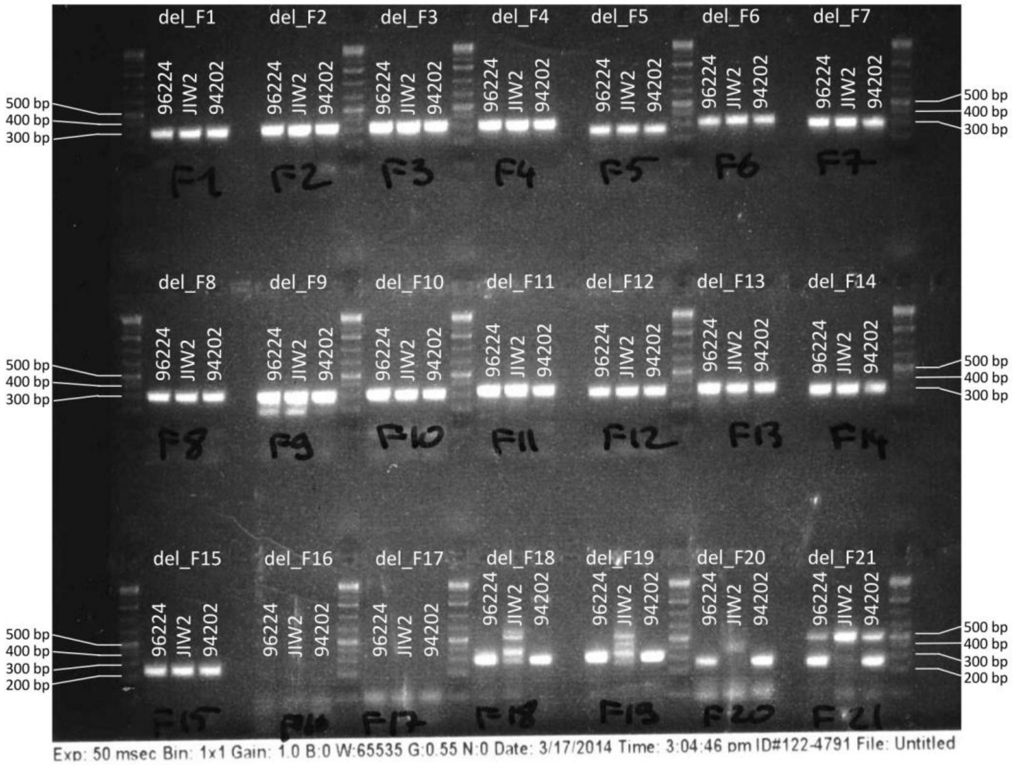

Fig. S6 Functional analysis of the interaction between *Pm2* and different family members and homologs in *B.g. triticale, B.g. secalis* and *B.g. hordei* in *N. benthamiana.* (a) A *Pm2* construct harboring two HA tags (C- and N- terminal) was co-expressed with *AvrPm2* but did not give HR. In the same assays, *AvrPm3*$^{a2/f2}$ was co-expressed with *Pm3a* as a positive control, and the hypersensitive cell death was weaker than the one resulting from co-infiltration of *AvrPm2* and *Pm2*. *AvrPm2* was also co-expressed with *Pm3a* which and did not result in HR. (b) The *AvrPm2* construct encoding for the full native protein with the signal peptide was co-expressed with *Pm2* and the observed hypersensitive response was weaker than in the infiltrations where the version without signal peptide was used. The *AvrPm2* family members *BgtE-5843* and *BgtE-5846* were also co-expressed with *Pm2* in *N. benthamiana* which did not result in HR. (c, d) Representative leaves from HR quantification assays (Fig. 3d) where the cell death response resulting from co-infiltrations of *AvrPm2* and *Pm2* constructs was compared to that resulting from co-infiltrations of *AvrPm3*$^{a2/f2}$ and *Pm3a* in 4:1 Avr:R ratio (c) and in 2:1 Avr:R ratio (d). (e) Co-expression of *Pm2* with the *AvrPm2* family member *BgtE-5842*, the direct homolog of *BgtE-5842* in rye mildew (*BgsE*-5842), and the closest homolog of *AvrPm2* in barley mildew (CSEP0066), did not result in HR. Co-expression of Pm2 with the *AvrPm2* haplotype found in the *B.g. triticale* isolate CAP-39-A1 (*BgtriticaleE-5845*) resulted in a hypersensitive cell death response. (f) The direct homolog of *AvrPm2* in rye mildew, *BgsE-5845* that differs from *AvrPm2* by only two amino acid polymorphisms, was co-expressed with *Pm2* and a strong HR was observed. The two variants containing either one of the two SNPs present in *BgsE-5845* (*BgtE-5845_T62I* and *BgtE-5845_R118C*) were also co-infiltrated with *Pm2* and HR was observed. Results in (a–f) were consistent across independent replicates from at least three experiments where four to eight leaves were assayed.

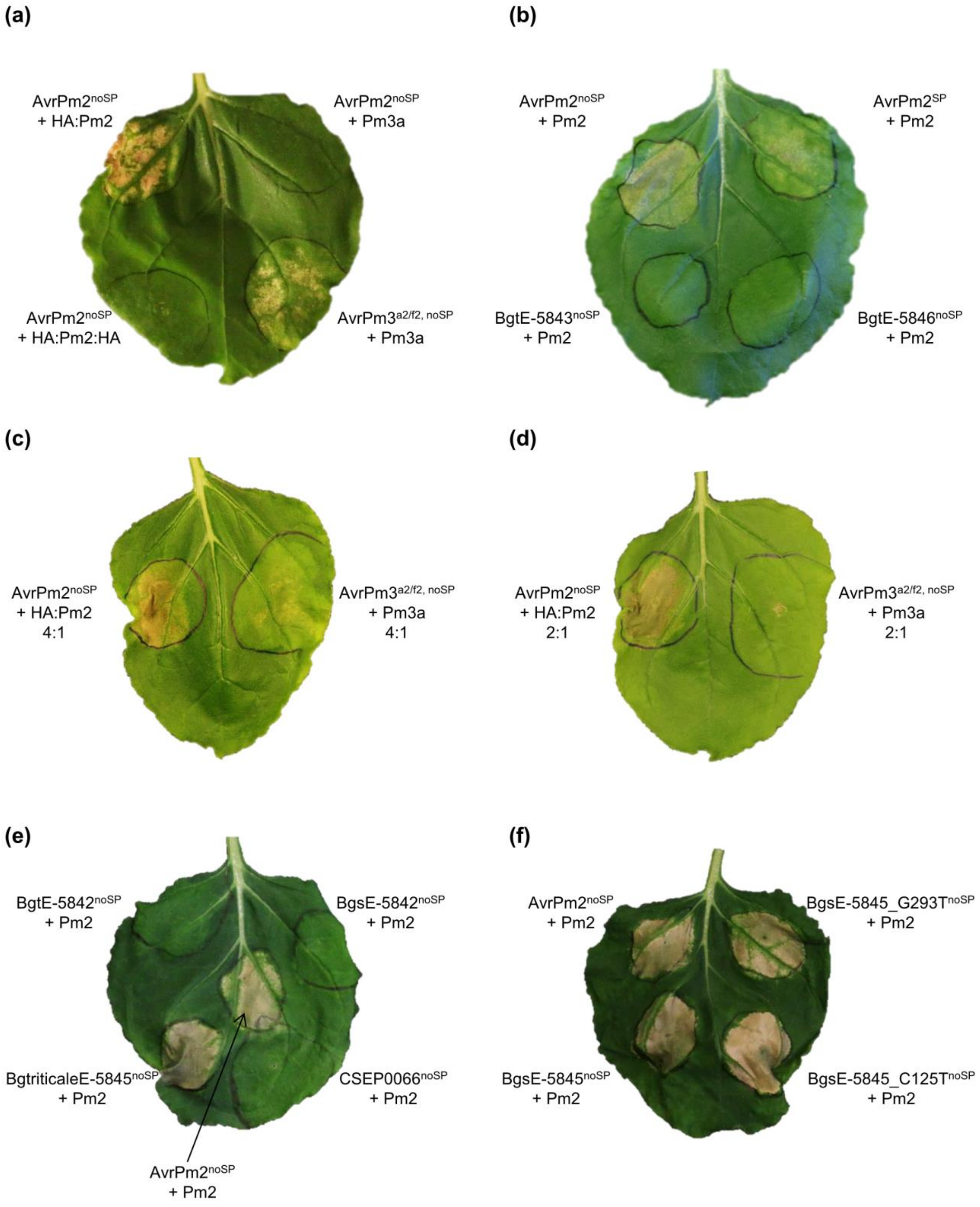
(a)
AvrPm2^{noSP} + HA:Pm2
AvrPm2^{noSP} + Pm3a
AvrPm2^{noSP} + HA:Pm2:HA
AvrPm3$^{a2/f2, noSP}$ + Pm3a
(b)
AvrPm2^{noSP} + Pm2
AvrPm2^{SP} + Pm2
BgtE-5843^{noSP} + Pm2
BgtE-5846^{noSP} + Pm2
(c)
AvrPm2^{noSP} + HA:Pm2 4:1
AvrPm3$^{a2/f2, noSP}$ + Pm3a 4:1
(d)
AvrPm2^{noSP} + HA:Pm2 2:1
AvrPm3$^{a2/f2, noSP}$ + Pm3a 2:1
(e)
BgtE-5842^{noSP} + Pm2
BgsE-5842^{noSP} + Pm2
BgtriticaleE-5845^{noSP} + Pm2
CSEP0066^{noSP} + Pm2
AvrPm2^{noSP} + Pm2
(f)
AvrPm2^{noSP} + Pm2
BgsE-5845_G293T^{noSP} + Pm2
BgsE-5845^{noSP} + Pm2
BgsE-5845_C125T^{noSP} + Pm2

Fig. S7 Gene expression of *AvrPm2* and *AvrPm3*$^{a2/f2}$ in the avirulent isolate 96224. Two additional biological replicates to Fig. 5 are represented here. The graphs show the mean normalized expression of *AvrPm2* (red line) and *AvrPm3*$^{a2/f2}$ (black line) in the avirulent parental isolate 96224, 1 to 5 days after infection of the susceptible wheat genotype Chinese Spring. Each data point is the average of three technical replicates. The error bars indicate the standard error of the mean (SEM).

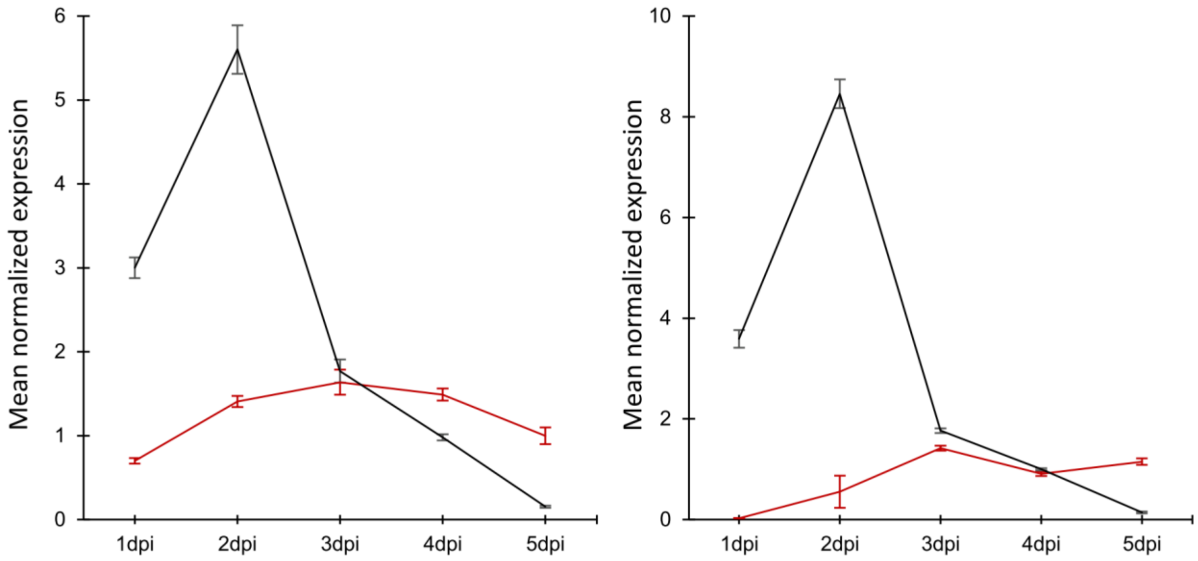

Fig. S8 Annotated protein alignment of the *AvrPm2* effector family in *B.g. tritici* and *B.g. hordei*. (a) Alignment of the 24 AVRPM2 family members in *B.g. tritici* and *B.g. hordei*. Protein sequences were aligned using Muscle 1:3.8.31-1 (Edgar, 2004) and visualized with ClustalX using the ClustalX Colour Scheme. The green box indicates the highly conserved signal peptide region and the green asterisks the predicted cleavage site. The conserved Y(x)xC motif and the conserved cysteine are indicated with a black box, and the conserved RxFP motif with a red box. The amino acids under positive selection are indicated with a red asterisk. (b) Representation of the conserved Y(x)xC motif in the 14 AVRPM2 family members in *B.g. tritici*. (c) Representation of the conserved RxFP motif in the 14 AVRPM2 family members in *B.g. tritici*.

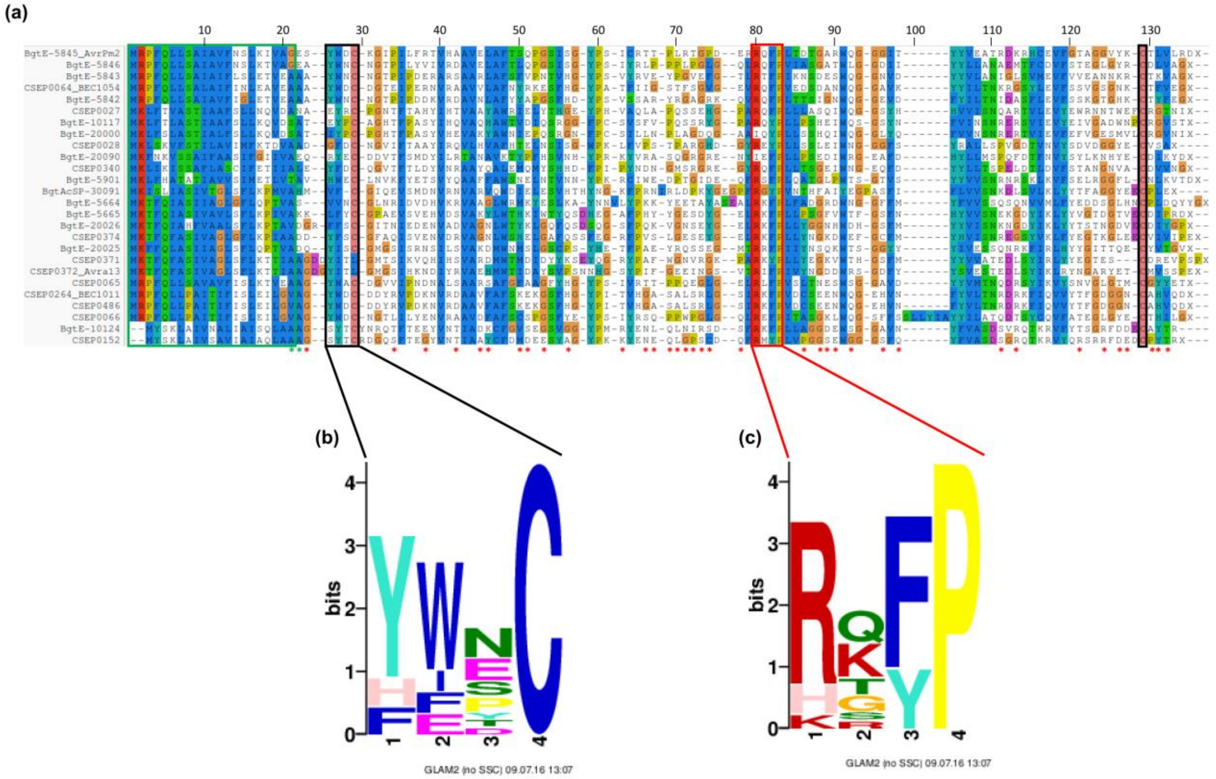

Fig. S9 Proposed molecular mechanism that could lead to the loss of the genomic region containing the *AvrPm2* gene. Homologous *Ino* retrotransposons flanking the *AvrPm2* region provide the template for unequal homogous recombination that results in two products: a recombinant locus carrying the deletion and a circular molecule that is subsequently degraded.

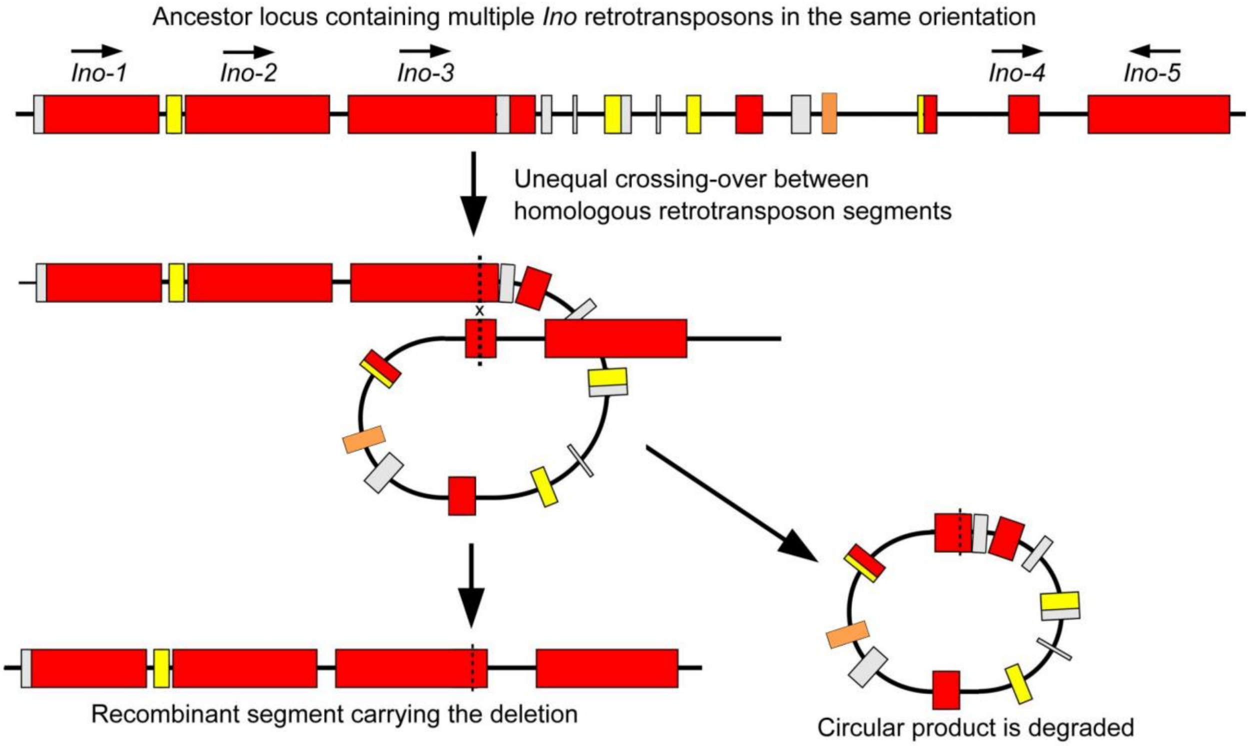

Table S1 Summary of the RNA samples used for qRT-PCR

ID	Isolate	Wheat cultivar	dpi	biological replicate	260/280[1]
1	96224	Chinese Spring	1	1	2.11
2	96224	Chinese Spring	1	2	2.02
3	96224	Chinese Spring	1	3	2.10
4	96224	Chinese Spring	2	1	2.13
5	96224	Chinese Spring	2	2	2.08
6	96224	Chinese Spring	2	3	2.04
7	96224	Chinese Spring	3	1	2.06
8	96224	Chinese Spring	3	2	2.15
9	96224	Chinese Spring	3	3	2.10
10	96224	Chinese Spring	4	1	2.08
11	96224	Chinese Spring	4	2	2.19
12	96224	Chinese Spring	4	3	2.08
13	96224	Chinese Spring	5	1	2.18
14	96224	Chinese Spring	5	2	2.14
15	96224	Chinese Spring	5	3	2.14

[1]The 260/280 ratio was measured by spectrophotometer (NanoDrop, Thermo Fisher Scientific).

Table S2 List of markers used for gene annotation, cloning and expression analysis

Marker	Fw/Rw	Sequence
5race5845_1[1]		AGACAAAAGTTGGAATGGTCTCA
3race5845_1[1]		TTTGGAACAGCAGGAGGTGTCT
5race5845_2[1]		TGTACGGCAAATTGAGGGGTAAC
3race5845_2[1]		TCTTTGGAACAGCAGGAGGTGTC
Pm2F[2]		CACCATGGCTGCCTCTGCCGCACTC
Pm2stopR[2]		CTAGTGCAACGACGACTCGGACATAAAT
Pm2HAstopR[2]		CTAAGCATAATCTGGAACATCGTATGGATAGTGCAACGACGACTCGGACATAAAT
HA::PM2F[2]		CACCATGTATCCATACGATGTTCCAGATTATGCTGCTGCCTCTGCCGCACTCGTT
5845topo[3]	Fw1	CACCATGAGACCATTCCAACTT
5845topo[3]	Rw1	CTAGTCTCGCAGAACTAATGTAC
5845topo[3]	Fw2	CACCATGAGACCATTCCAACTTTTG
5845topo[3]	Rw2	CTAGTCTCGCAGAACTAATGTACAT
E5846_topo[3]	Fw1	CACCATGAGACCATTCCAACTTTTGTCTGCT
E5846_topo[3]	Rw1	CTAGCCCGCAACTTTCGTACA
E5846_topo[3]	Fw2	CACCATGAGACCATTCCAACTTTTGTCTGCTATTG
E5846_topo[3]	Rw2	CTAGCCCGCAACTTTCGTACACCTTTT
E5846_0SP_topo[3]	Fw1	CACCATGGCAGCTTATTGGAATTGCAATGGTACTCCA
E5846_0SP_topo[3]	Fw2	CACCATGGCAGCTTATTGGAATTGCAATGGTACT
E5842_topo[3]	Fw1	CACCATGAGACCTTTCCAACTTTTGTCTGCTATT
E5842_topo[3]	Rw1	CTAGCCTTCAAAATATGTACACTTATGGCCAGT
E5842_topo[3]	Fw2	CACCATGAGACCTTTCCAACTTTTGTCTGCT
E5842_topo[3]	Rw2	CTAGCCTTCAAAATATGTACACTTATGGCCA
E5842_0SP_topo[3]	Fw1	CACCATGGCAGCTTATTGGAATTGTAATGGTACTCCA
E5842_0SP_topo[3]	Fw2	CACCATGGCAGCTTATTGGAATTGTAATGGTACTCCAATT
E5843_topo[3]	Fw1	CACCATGAGACCATTCCAACTTTTGTCTGCTATT
E5843_topo[3]	Rw1	CTAGCCGGCAACTAAATCACATCTATAGC
E5843_topo[3]	Fw2	CACCATGAGACCATTCCAACTTTTGTCTGCTATTGC
E5843_topo[3]	Rw2	CTAGCCGGCAACTAAATCACATCTATAG
E5843_0SP_topo[3]	Fw1	CACCATGGAAGCTTATTGGAATTGTAATGGTACTCCAATT
E5843_0SP_topo[3]	Fw2	CACCATGGAAGCTTATTGGAATTGTAATGGTACTCCA
E5845_int_qPCR[4]	Fw1[5]	CGAACTGTCCATGCTGCGGTGG
E5845_int_qPCR[4]	Rw1[5]	CCTCCTGCTGTTCCAAAGACTTCGC
E5845_int_qPCR[4]	Fw2[6]	ACGGACTGGACCAGACGAACGT
E5845_int_qPCR[4]	Rw2[6]	TCCTGCTGTTCCAAACACTTCGCAAT

[1]Markers used to annotate BgtE-5845.

[2]Markers used for cloning of Pm2.

[3]Markers used for cloning of *B.g tritici* genes.

[4]Markers for RT-qPCR.

Table S3 List of markers used for genetic mapping

Marker	Fw/Rw	Sequence	Restritcion enzyme
BgtE-5842[1]	Fw1	CGAACCTTGCCAGTTTCCAA	
BgtE-5842[1]	Rw1	GGACAAACTTCCTGCCACTCA	
BgtE-5842[1]	Fw2	TTTCTTCCAGCCCCTCGATA	
BgtE-5842[1]	Fw3	GGCCAAATTTACTGCATCTCG	
BgtE-5842[1]	Rw3	GCAAACAGCTTTGTCCCTCA	
BgtE-5842[1]	Fw4	GCTGCAGCCTCTACTGTCTCC	
BgtE-5842[1]	Rw4	TTCCTCCCCAATCTGCAAAC	
Bgt-2686[1]	Fw1	GTTTCCTGTCGCCCTTCAGCT	
Bgt-2686[1]	Rw1	ACAACCCCTTCTCAACGGGC	
Bgt-2686[1]	Fw2	AACTGCTAGCCCCGGCACAT	
Bgt-2686[1]	Rw2	CCCACGCTAACATGATGCGC	
Bgt-2686[1]	Fw3	ACTCTGACACAGACTCAACACGCAG	
Bgt-2686[1]	Rw3	TCCAGCGCCCTTACCCTCGCTTC	
F25[2]	Fw	CATCTGTGGATCGAGGCGCTTT	*RsaI*
F25[2]	Rw	CCCTCATGTATCTTCTCACACGATG	*RsaI*
F2[3]	Fw1	GGACTATTTCGGAGCACCAC	
F2[3]	Rw1	GGTTCCTGGGGGAATAAAAT	
F2[3]	Fw2	ACCTTCTTGCAAACGCAGAAATGA	
F2[3]	Rw2	AGTGAACCGTCCTGTCTTTCTCT	
F2[3]	Fw3	ATGTGATTTAGTTGCCGGCTAGG	
F2[3]	Rw3	AGTGAACCGTCCTGTCTTTCTCTCTA	
F5[3]	Fw	GGACGAGCAGCCATCAATA	
F5[3]	Rw	CGCCTGTCCAGCCTAATTT	
F7[3]	Fw	CCCAGCTACCACATTGGA	
F7[3]	Rw	GGAATACCTATGGCAAGCATAA	
F11[3]	Fw1	GCAGCTAAAGGGCCATAACC	
F11[3]	Rw1	AAGCTTTGGCGTCTCATGTC	
F11[3]	Fw2	AGCAGCTAAAGGGCCATAAC	
F11[3]	Rw2	CCCCCATAACCTGTTGTCC	
F12[3]	Fw1	TGTTGAAATATGACACATCTTGG	
F12[3]	Rw1	TGCACGCTAACAAAACAACC	
F12[3]	Rw2	CCGTGCACGCTAACAAAAC	
F18[3]	Fw1	GGACAAACTTTCTGCCACTCA	
F18[3]	Rw1	AACCTTGCCAGCTCTCATCA	
F18[3]	Fw2	CTCCAGGAGCACCACCATAA	
E5845Int1[4]	Fw	TGAGACCATTCCAACTTTTGTCT	
E5845Int1[4]	Rw	AGACACCTCCTGCTGTTCCAAA	
E5845Int2[4]	Fw	GTTACCCCTCAATTTGCCGTACA	
E5845Int2[4]	Rw	GACACCTCCTGCTGTTCCAAAGA	
E5845_100[4]	Fw	CACAAACTTTCTGCCACTCAAA	
E5845_100[4]	Rw	ATAGACACCTCCTGCTGTTCCAA	
E5845_d3[4]	Fw	CGACTCGATGTACTTCGGACCA	
E5845_d3[4]	Rw	TGCGCCATGTGGCCTAAAAA	

[1]Markers designed on SNPs in or around genes identified by the collinearity approach.
[2]CAPS markers used for genetic mapping (the corresponding restriction enzyme is indicated on the right).

[3]Markers designed on SNPs tested by sequencing for genetic mapping.

[4]Presence/absence genetic markers designed in and around BgtE-5845 and used for validating genetic co-segregation with AvrPm2.

Table S4 BAC end markers. List of BAC end markers used to test the BAC overlap. BAC end markers were designed based on the BAC end sequences of the Fingerprint Contig (FPC) assembly.

Marker	Fw/Rw	Sequence
8e01f_F1	Fw	GCGTCCAAGTTTAGCGGGCA
8e01f_F1	Rw	CTTCTTGACCTGTCCGGCCC
8e01f_F2	Fw	TGACTGGACCGATCCCAGACA
8e01f_F2	Rw	AACAGGTTATGAGCCCGGCG
21d24f_F1	Fw	GTAGCTTGTTCCCGGGCTGG
21d24f_F1	Rw	CACGTCCTCCCCGTCTACCA
21d24f_F2	Fw	CCTCGTGGGCCGACTTCTTC
21d24f_F2	Rw	GGTGTCCGAGATGGGCCAAG
3b20f_F1	Fw	CGCTGGACCCAAAAACATGGC
3b20f_F1	Rw	AGATAGCGGCTCCCCCTCAA
3b20f_F2	Fw	TGAGGGGGAGCCGCTATCTT
3b20f_F2	Rw	TGTTGACTGTCTGACGCCGT
11j01f_F1	Fw	GGGTGGGTTCACGGTGACAG
11j01f_F1	Rw	GCGAGAAGCTGGCGTACTGT
11j01f_F2	Fw	TGACTGTACCAATGAGAAGCGGT
11j01f_F2	Rw	CGCTTGACCCAACCCTCCAG
23f12f_F1	Fw	TTGCCACCTCCCTGTCGTCT
23f12f_F1	Rw	TGCAGTCACGGATTGGGAGC
23f12f_F2	Fw	ACCCAGCTCAAAGTTGAAAGGA
23f12f_F2	Rw	CCACGATAAGAGAAGCATGGCGA

New Phytologist

Table S5 Markers used for estimating the size of the deletion. List of markers designed every 500 bp over 20 kb around BgtE-5845 in order to delimit the borders of the deletion.

Marker	Fw/Rw	Sequence	Name	Fw/Rw	Sequence
del_F1	Fw	GCCCAGTACTAGCCATTCCA	del_F20	Rw	CCCAGCTGACCAGACACAAA
del_F1	Rw	ACACATTGCCCTCCGAAACT	del_F21	Fw	CACCTAGTGGAGCGAGTTGA
del_F2	Fw	AGGTTTCTGTTGCAGCGAGA	del_F21	Rw	TGTCAACCTGAGGTCAGTCG
del_F2	Rw	AGAACATTTCGAGGCAGCCA	del_F22	Fw	CTGCCATGCCAATTCCATGT
del_F3	Fw	TGCTTGGCTTGGAGATGTGT	del_F22	Rw	CCGAAGTACATCGAGTCGGT
del_F3	Rw	TAAACAGGCAGCTGCGCTAT	del_F23	Fw	GAGCTCTGAAGGCGAGATCC
del_F4	Fw	CCGATCTTGGAGTTGGAGCA	del_F23	Rw	CCTGACTGTAGTAGCCGCTC
del_F4	Rw	ACAGGCCCAGAAGCTAAACA	del_F24	Fw	TTGCTGGGCTGAAAACTCCA
del_F5	Fw	TTTTCTGTCCAGCGATGGGG	del_F24	Rw	GGACAATCACGTAGGCCACA
del_F5	Rw	AGGGAGTATACGCTGGCAGA	del_F25	Fw	CTAGCTTTGACTCCAGGGGG
del_F6	Fw	TCTGGAGAGGTGCGAGATGA	del_F25	Rw	ACACTGGTGATTGTCGGAGG
del_F6	Rw	AGCAGAGATGGGGTGGTTTG	del_F26	Fw	TCGGGATTGCGATGGATAGC
del_F7	Fw	TCGGAGCCCCCATCTCTAAA	del_F26	Rw	TAACGAGCCACCACTGCATT
del_F7	Rw	AGACTCACACCAAACGAGCC	del_F27	Fw	TTACTTCGCTCCCGTGTTCC
del_F8	Fw	ACATGTCTCGCAAGTGCTGA	del_F27	Rw	ATCCGCCAATTCACAGACGT
del_F8	Rw	AAATAGCTCGCCTCGTTCGT	del_F28	Fw	AACAGCAGGGTGATCTCCAA
del_F9	Fw	GATGCGAGGCACCAGTATCA	del_F28	Rw	TCTCGTCCCCCTCCTTCATT
del_F9	Rw	AATGGAACCAGGCCCAGATG	del_F29	Fw	CGCAGCAGTCAAAGCTTCTG
del_F10	Fw	CTGCTTTGCTGTACAGTGCC	del_F29	Rw	ACAGAGCTGAATCACTCGTGG
del_F10	Rw	AAGCTAGCTCGACGCAAAGT	del_F30	Fw	TCCCATGCCACACCTTGTAC
del_F11	Fw	GCCCGGGCTCATAACCTATT	del_F30	Rw	CACCTGATATGGTCCCGGTG
del_F11	Rw	ATGGCTGAATGGTTGGCAGT	del_F31	Fw	TGGTGATAGAGGGCTGTGGT
del_F12	Fw	ACCCCTCCTGAGTCTGACTC	del_F31	Rw	GATTCCGCACGACAACTTGG
del_F12	Rw	TTGCGATCTGTAGCAGGCAA	del_F32	Fw	GCCACTCAAATACAGCCAGC
del_F13	Fw	GTGTTTCGAGTGCTGCAGTG	del_F32	Rw	TCTGGTCCAGTCCGTAGAGG
del_F13	Rw	GGGATCCGTCAAGTCAACGT	del_F33	Fw	TCACTGTGCCTGTTACGACC
del_F14	Fw	CCGGGCTCATAACCTGTTGT	del_F33	Rw	AACATGGGGTCTAAAGGGCG
del_F14	Rw	TGCTCCTGCTTCGTTGAGTT	del_F34	Fw	GAACAGCTTTCCTGCATGCA
del_F15	Fw	CCCGCTGGGTTATGGAACTT	del_F34	Rw	CCCGCTCAAAGACTTCACGA
del_F15	Rw	GCTGCTCCTTGACATCTTCG	del_F35	Fw	TCTGGCAAGCAAGAACTCGA
del_F16	Fw	TTTGGCGCATTTGTTCAGGG	del_F35	Rw	GCTTCGGGAGTTACTCGCAT
del_F16	Rw	ATTCAGCTGCGCAACTGTTG	del_F36	Fw	GAAGGTGTCATGGTGGCTCA
del_F17	Fw	TCTGCTCTGATGCCGCATAG	del_F36	Rw	CCACCTCTAACGTACTGGCG
del_F17	Rw	TGAAAGCGAGCTTTTTGGCC	del_F37	Fw	CTATCAAGGGCGGACACTGG
del_F18	Fw	AGGAGTCGTTACCAACAGGG	del_F37	Rw	TCCGTGGTACTGTCTTGGGT
del_F18	Rw	TGCCAGCCGGAATTAATCGT	del_F38	Fw	GCTGAAGCAAGAGCCCAAGA
del_F19	Fw	TGCGTTTGCTCTGTTTGCTC	del_F38	Rw	ACACAGCTCCTATGCCCAAC
del_F19	Rw	GGGACGCCTTGCAATTTGTT	del_F39	Fw	GGAACACGGGAGCGAAGTAA
del_F20	Fw	AGAAGTATTGGCACGTCGGG	del_F39	Rw	GTGGCTCGTTACAGATGCGA

Table S6 Whole genome resequencing strategies for the 22 Chinese isolates.

Isolate	Company	Strategy
1-25	Novogene	PE100
2-25	BGI	PE90
46-30	BGI	PE90
46-31	BGI	PE90
48-18	BGI	PE90
3-53	Novogene	PE100
5-83	Novogene	PE100
6-69	Novogene	PE100
7-8	Novogene	PE100
10-8	Novogene	PE100
12-50	Novogene	PE100
NZ-1	Novogene	PE100
17-40	Novogene	PE100
44-3	Novogene	PE100
45-10	Novogene	PE100
46-25	Novogene	PE100
SD-5	Novogene	PE100
36-70	Novogene	PE100
45-6	Novogene	PE100
47-3	Novogene	PE100
52-27	BGI	PE90
15-9	Novogene	PE100

Table S7 List of the *B.g. tritici* and *B.g. triticale* isolates used for genome-wide association studies for the identification of *AvrPm2*. Table headers indicate the geographical origin of the isolate (1st column), the forma specialis it belongs to (2nd column), isolate ID (3rd column), the year it was collected (4th column). The phenotype on Pm2 wheat as well as the presence/absence of AvrPm2 are indicated in columns 6 and 7, respectively.

Table S7 is available as a separate file.

Table S8 Genetic map of 96224 x JIW2 mapping population based on KASP markers. The 96224 x JIW2 genetic linkage map consists of 248 Kompetitive allele Specific PCR (KASPar) markers arranged in 26 linkage groups covering 1,275 cM (19 linkage groups with at least 4 markers). Linkage was considered significant at a threshold of 30 cM. The AvrPm2 locus is located on linkage group 15 at 7.0 cM from marker 051_LE. Linkage groups are numbered according to their length (in cM) and are not related to chromosomes. The distance between each marker is given in cM.

Table S8 is available as a separate file.

Table S9 Blast hits of Pm2 against wheat and barley databases

Databank	Contig	Score	Identities	Percentage	Expressed[3]
IWGSC - 5DS[1]	contig_2771130	6442	3668/3731	98	yes
IWGSC - 5DS[1]	contig_2760423	3709	3106/3797	82	no
IWGSC - 5DS[1]	contig_2740054	2066	1801/2226	81	no
IWGSC - 5AS[1]	contig_154836	5988	3566/3743	95	no
IWGSC - 5BS[1]	contig_2290994	3647	2600/2982	87	no
IWGSC - 5BS[1]	contig_2266554	3523	3018/3719	81	no
Barley high confidence genes[2]	MLOC_11605.2	1235	699/1299	53	

[1]Indicates the BLAST hits for the Pm2 nucleotide sequence (3,834 nucleotides) against the Wheat Sequence Survey Assembly database.

[2]Indicates the best hit of the PM2 protein sequence (1,278 amino acids) against the barley High Confidence gene database is indicated.

[3]A gene was considered expressed if it was supported by transcripts in the Generic Genome Browser version 2.33 of the Wheat IWGSC survey sequence annotation (https://urgi.versailles.inra.fr/gb2/gbrowse/wheat_survey_sequence_annotation/).

Table S10 Results of the ANOVA for HR intensity in HR quantification assays. We performed ANOVA for the measurements of HR intensity obtained by fluorescence imaging of the infiltrated *N. benthamiana* leaves.

	Df	Sum Sq	Mean Sq	F value	Pr(>F)	
Gene[1]	1	142878	142878	161.815	4.82E-11	***
Ratio[2]	1	11102	11102	12.5734	0.002028	**
Gene:Ratio	1	111	111	0.1253	0.727062	
Residuals	20	17659	883			

[1]Genes are either the AvrPm2-Pm2 or the AvrPm3a2/f2-Pm3a interaction

[2]Ratios are either 4:1 or 2:1 Avr:R ratio.

Table S11 Results of the TukeyHSD post-hoc test. A TukeyHSD post-hoc test was conducted on the ANOVA results presented in Supporting Information Table S10. Measurements were grouped according to interaction (Pm2 for AvrPm2-Pm2 and Pm3a for AvrPm3a2/f2-Pm3a) and to Avr:R ratio (A for 4:1 and B for 2:1). Differences are considered significant if the adjusted *P*-value (*P* adj) is < 0.01.

Comparison	diff[1]	lwr[2]	upr[3]	*P* adj[4]
Pm3a-Pm2	-154.3146000	-179.6195000	-129.0097000	0.0000000
B-A	-43.0154200	-68.3202900	-17.7105400	0.0020276
Pm3a:A-Pm2:A	-150.0205000	-198.0386600	-102.0023392	0.0000002
Pm2:B-Pm2:A	-38.7213300	-86.7394900	9.2968274	0.1422075
Pm3a:B-Pm2:A	-197.3300000	-245.3481600	-149.3118392	0.0000000
Pm2:B-Pm3a:A	111.29917	63.28101	159.3173274	0.0000141
Pm3a:B-Pm3a:A	-47.3095	-95.32766	0.7086608	0.0543866
Pm3a:B-Pm2:B	-158.60867	-206.62683	-110.5905059	0.0000001

[1]Indicates the difference between the mean of the two groups.

[2]Indicates the lower bound of the 95% confidential interval.

[3]Indicates the upper bound of the 95% confidential interval.

[4]Indicates the adjusted p-value.

Table S12 Signal peptide and disulfide bond predictions in the AvrPm2 family in *B.g. tritici*

	Signal peptide		Disulfide bound	
	Prediction[1]	Cleavage site[2]	Prediction[1]	Bound[2]
BgtAcSP-30091	Y	21-22	Y	26-118
BgtE-10117	Y	21-22	Y	57-114
BgtE-10124	Y	20-21	Y	25-113
BgtE-20000	Y	21-22	Y	57-114
BgtE-20025	Y	21-22	N	-
BgtE-20026	Y	23-24	Y	28-116
BgtE-20090	Y	21-22	Y	27-113
BgtE-5664	Y	21-22	Y	26-116
BgtE-5665	Y	21-22	Y	27-115
BgtE-5842	Y	21-22	Y	27-113
BgtE-5843	Y	21-22	Y	37-113
BgtE-5845	Y	21-22	Y	27-59
BgtE-5846	Y	21-22	N	-
BgtE-5901	Y	22-23	Y	59-113

[1]Indicates if a signal peptide and a disulfide bond are predicted (Y) or not (N) respectively.
[2]Indicates, the position of the signal peptide and disulfide bond respectively, if predicted.

Table S13 Structure prediction of the AVRPM2 in *B.g. tritici* and *B.g. hordei*. Summary of 3D protein structure predictions for the AVRPM2 effector family in *B.g. tritici* and *B.g. hordei* as obtained from RaptorX (http://raptorx.uchicago.edu/) (Källberg *et al*., 2012).

AvrPm2 Family	Best Template[1]	Score[2]	*P*-value[3]	uGDT(GDT)[4]	Size[5]
BgtAcSP-30091	1bu4A	31	3.34E-03	42 (42)	101
BgtE-10117	1rdsA	42	3.38E-05	55(56)	99
BgtE-10124	1rdsA	32	5.28E-04	49 (50)	98
BgtE-20000	1rdsA	47	5.49E-05	55 (56)	99
BgtE-20025	1rdsA	38	9.56E-04	54 (55)	98
BgtE-20026	1bu4A	38	1.02E-03	49 (50)	99
BgtE-20090	1rdsA	51	1.30E-05	54 (55)	98
BgtE-5664	3whoA	36	9.39E-04	50 (48)	103
BgtE-5665	3whoA	40	3.65E-04	50(50)	100
BgtE-5842	1rdsA	46	8.50E-05	55 (56)	98
BgtE-5843	1rdsA	50	4.83E-05	54 (55)	98
BgtE-5845 - AvrPm2	1rdsA	50	1.09E-04	56 (56)	99
BgtE-5846	1rdsA	52	2.25E-05	56 (57)	98
BgtE-5901	1rdsA	49	1.06E-04	56(57)	98
CSEP0027	1rdsA	49	2.81E-05	55 (56)	99
CSEP0028	1rdsA	53	1.88E-05	54 (57)	95
CSEP0064	1rdsA	50	1.11E-04	55 (56)	98
CSEP0065	1rdsA	50	4.25E-05	55 (56)	98
CSEP0066	1rdsA	49	8.35E-05	52 (51)	102
CSEP0152	1bu4A	34	6.65E-04	51 (52)	98
CSEP0264	1rdsA	46	9.54E-05	55 (58)	96
CSEP0340	1rdsA	52	4.16E-05	55 (56)	98
CSEP0371	1bu4A	32	1.94E-03	50 (50)	101
CSEP0372 - Avr_{a13}	1fusA	35	1.18E-03	50 (49)	102
CSEP0374	1rdsA	37	1.42E-03	49 (48)	101
CSEP0486	1rdsA	46	9.54E-05	55 (58)	96

[1]Indicates the best template for 3D structural modelling.
[2]Indicates the alignment score falling between 0 and the sequence length.
[3]Indicates the likelihood of a predicted model being worse than the best of a set of randomly-generated models for this protein. For mainly alpha helixes, *P*-value less than 10^-3 is a good indicator. For manly beta sheets, *P*-value less than 10^-4 is a good indicator

[4]Indicates uGDT and GDT where uGDT is the unnormalized GDT (Global Distance Test) score defined as 1*N(1)+0.75*N(2)+0.5*N(4)+0.25*N(8), where N(x) is the number of residues with estimated modeling error (in Å) smaller than x. GDT is calculated as uGDT divided by the protein length and multiplied by a 100. For a protein with >100 residues, uGDT>50 is a good indicator. For a protein with <100 residues, GDT>50 is a good indicator.

[5]Is the length of the protein in amino acids.

Methods S1 BAC selection and sequencing

The *B.g. tritici* BAC library was previously produced from isolate 96224 as described by Oberhaensli and colleagues (2011). BACs were selected based on their positions in the BAC fingerprint contig (FPC) and in the linear topological contig (LTC) assemblies (Frenkel *et al.*, 2010). 3D-DNA pools of the library were screened by PCR using genetic and physical markers. Candidate clones were confirmed by PCR and plasmids were extracted using the Qiagen Large-Construct Kit according to the manufacturer's protocol. BAC insert sizes were estimated by digestion with *Not*I and pulsed field gel electrophoresis. Selected BAC clones were sequenced using Illumina MiSeq technology (2 × 250 bp paired end; GATC Biotech). Individual BAC reads were assembled using the CLC Genomics Workbench version 6.0.1. Each BAC was assembled individually with two different sets of parameters as follows: (i) assembly 1: distance between paired reads = 1 to 700 bp, word size = 64bp, minimum contig length = 2000 bp; and (ii) assembly 2: distance between paired reads = 100 to 350bp, word size = 19bp, minimum contig length = 1000 bp. Assembly 1 resulted in large contigs that were used to order the smaller contigs obtained from assembly 2. The *AvrPm2* assembled locus was integrated into contig 51 of the reference genome (Wicker *et al.*, 2013). The physical markers used to confirm the BAC assembly are listed in Table S7.

Methods S2 Illumina whole-genome resequencing

Genomic DNA of 22 Chinese isolates selected for resequencing was extracted from fresh conidiospores using a CTAB method (Ristaino *et al.*, 1998) with minor modifications. Briefly, 240 mg conidia in 700 µl extraction buffer was vortexed with glass beads for 3 min. 40 µl 20% SDS, 65 µl 500 µg ml^{-1} proteinase K and 2 µl 500 µg ml^{-1} RNase was added and incubated at 65°C for 1 h to digest proteins and RNA. Then 700 µl phenol-chloroform was added and mixed followed by centrifugation at 13,201 ***g*** for 10 min. DNA in the supernatant was precipitated with isopropanol overnight at -20°C and then collected with centrifugation at 12,000 rpm for 10 min. The pellets were then washed with 70% ethanol twice, DNA was dissolved in 100 µl TE solution and then stored at -80℃. The concentration, integrity and purity of DNA samples were evaluated with a Qubit Fluorometer (Invitrogen) followed by agarose gel electrophoresis.

DNA library construction and sequencing was carried out at BGI (Shenzhen, China) for 5 isolates and at Novogene (Beijing, China) for 17 isolates in 2012 and in 2014, respectively (Table S9). Sequencing libraries for these isolates were built using a DNA Library Prep Kit (NEBNext® Ultra™) according to the standard procedure provided by the manufacturer. About 1 µg of genomic DNA was sheared and approximately 500 bp length were selected for Illumina library preparation. The adapter ligated DNA was enriched by several rounds of PCR amplification. Finally, Agilent® Bioanalyzer 2100 was used to detect the library distribution and Q-PCR was employed to determine the library concentration. The libraries were sequenced on an Illumina HiSeq 2000 platform to generate pair-end reads with at least 4 Gb data per sample. Paired-end 90 (PE90) and paired-end 100 (PE100) sequencing strategies were employed by BGI and Novogene, respectively (Table S9).

Methods S3 RNA extraction and qRT-PCR assays

Samples of susceptible wheat leaf segments (cv 'Chinese Spring') infected with the isolates 96224 were collected every 24 h and frozen in liquid nitrogen. We performed three individual infections corresponding to the three biological replicates. The samples were grinded and total RNA was extracted using the Qiagen miRNeasy Mini Kit (50) according to manufacturer's instructions. RNA was quantified using a spectrophotometer (NanoDrop, Thermo Fisher Scientific). RNA quality was assessed by gel electrophoresis and based on the 260/280 ratios obtained with the spectrophotometer (Supporting Information Table S1). Samples with a ratio 2.0 – 2.2 were used for cDNA synthesis. All RNA samples were stored at -80°C until cDNA synthesis, usually the day after extraction. RT-qPCR ready cDNA was synthesized from 1.5 µg total RNA using the iScript cDNA synthesis kit (Bio-Rad) according to the manufacturer's instructions.

We have used Glyceraldehyde 3-phosphate dehydrogenase (Gapdh) and Actin (Act1), two commonly known housekeeping genes in fungi. Both genes were previously validated as stable internal controls for gene expression studies in *Blumeria graminis* by Bourras *et al*. (2015)and Pennington *et al*. (2016). All our gene expression data is normalized to Gapdh, in consistence with previous work in wheat powdery mildew (Bourras *et al.*, 2015). For *AvrPm3*$^{a2/f2}$, we used the primers published in Bourras *et al*. (2015). Primers for *AvrPm2* were designed after in silico testing by blast searches against the *Blumeria graminis* f.sp *tritici*. The length of the amplicon is 132 bp (see Supporting Information Table S2). Primer specificity was additionally tested by gel electrophoresis analysis of PCR amplicons from genomic DNA and cDNA. Primer efficiencies were tested with series of 4 dilutions (1x, 4x, 16x and 64x) using the Bio-Rad Real-Time PCR CFX Manager Software version 3.5 and the curve for *AvrPm2* primer efficiency is available in Supporting information Fig. S1.

We used the SsoFast EvaGreen Supermix chemistry (BioRad) according the manufacturer's instructions. Each reaction was done in a 10 µl volume, with 4 µl of qRT-PCR ready cDNA, and a final primer concentration of 500 nM. We used 10 µl of final reaction volume. All assays were done in a BioRad C1000 Touch thermal cycler, equipped with the CFX96 Real-Time PCR System, with the following program:

1. 95.0 C for 0:30
2. 95.0 C for 0:03
3. 60.0 C for 0:05 + Plate Read
4. GOTO 2, 39 more times
5. Melt Curve 65.0 to 95.0 C, increment 0.5 C, 0:05 + Plate Read
6. END

For each biological replicate we performed three technical repetitions and gene expression analysis was performed using the Bio-Rad Real-Time PCR CFX Manager Software version 3.5, and the ddCq formulae, according to the manufacturer's instructions.

Methods S4 Genome-wide effector annotation and *AvrPm2* family identification in different *formae speciales*

To identify the *AvrPm2* effector family we performed a *de novo* annotation of the *B.g. tritici* genome and redefined the predictions of effector families. We also assembled *de novo* the *B.g. tritici* transcriptome (Wicker *et al.*, 2013) with CLC-workbench 6 with standard parameters. Transcriptome assembly resulted in 16,440 contigs which were used subsequently. We used the *B.g. tritici* gene database (Wicker *et al.*, 2013) as query in blast searches against the *B.g. tritici* genome and kept all hits with at least 50 amino acids in length and 20% identity at the protein level. If no genes were previously annotated in the aligned region we considered an additional window of ± 500 bp up- and downstream. In a second step we blasted every window against the transcriptome and kept hits with more that 95% identity at the nucleotide level. If the alignment had an open reading frame (ORF) we extended the sequence, using the transcriptome as template, until the closest start and stop codons. If in the 5’ direction, we found a stop codon before finding a start codon we retained the original aligned sequence without extending it in the 5’ direction. Results were further parsed to avoid redundant annotations. To exclude repetitive elements we used every new gene as query in blast search against the *Blumeria* and *Triticeae* transposable elements (TE) databases (Bg +PTREP12) (Wicker *et al.*, 2013), http://wheat.pw.usda.gov/ITMI/Repeats/, accessed 01/03/2014), and hits with blastp e-value ≤ 10^{-5} were excluded. This annotation produced a large number of gene fragments. We retained only ORFs longer than 50 amino acids and we then performed further manual curation excluding all the genes that could be aligned more than 10 times in the genome by gmap (version 2013-07-20) (Wu & Nacu, 2010). This process was iterated 9 times with the newly identified genes. The protein databases of *Podosposra anserina*, *Neurospora crassa* and *B.g. hordei* were downloaded from http://podospora.igmors.u-psud.fr/download.php, www.broadinstitute.org/annotation/genome/neurospora/MultiDownloads.html and http://www.blugen.org/ (accessed 01/03/2014). After elimination of proteins with homology in the repeat databases (Bg +PTREP12, blastp e-value ≤ 10^{-5}) we retained 10,604 proteins *for P. anserina*, 9,733 for *N. crassa*, and 6,011 for *B.g. hordei.*

To cluster proteins in families we performed “all against all” blast searches using proteins sequences form *B.g. hordei*, *B.g. tritici*, *N. crassa* and *P. anserina*. Hits with e-value greater than 0.001 and alignment length shorter than 70% of the query length were excluded. Protein families

were generated with the markov cluster algorithm implemented in the MCL software (with option -I 6) (Enright *et al.*, 2002). We then defined secreted genes as all genes which encode a protein with a predicted signal peptide but do not have a trans-membrane domain (predicted with signalp 4.1) (Petersen *et al.*, 2011). Finally, we defined as candidate secreted effector proteins (CSEPs) all proteins belonging to families composed exclusively of *B.g. tritici* and *B.g. hordei* proteins with at least one secreted gene. With this pipeline we identified 1,189 CSEPs (597 in *B.g. tritici* and 592 in *B.g. hordei*). Here we used a different approach to predict mildew CSEPs compared to Pedersen *et al.* (2012) which has resulted in the identification of a larger set of effectors in *B.g. tritici* and *B.g. hordei*. In this annotation we have also included genes lacking a predictable signal peptide but showing homology to actual CSEPs. We than re-clustered these 1,189 CSEPs with the same pipeline (with option -I 1.4) and found that 1,123 belonged to a family of at least 2 proteins. We then selected the family containing *BgtE-5845*. To identify haplotypes of *AvrPm2* in the different *B.g. tritici* and *B.g.triticale* isolates, we ran a SNP call on the genomic mapping using vcftools (Danecek *et al.*, 2011). We considered as high-confidence SNPs all positions with a minimum mapping score of 20, a minimum coverage of 8×, a minimum frequency of the alternative call of 0.9 and < 5 missing genotypes. To identify the family members in *B.g. secalis* we mapped the *B.g. tritici* family members against the *B.g. secalis* assembly (Menardo *et al.*, 2016) using gmap (version 2013-07-20) (Wu & Nacu, 2010).

Methods S5 *In silico* analysis of proteins encoded by *Pm2* and *AvrPm2*

In silico identification and annotation of conserved protein domains in the PM2 sequence was done with i) COILS (http://www.ch.embnet.org/software/COILS_form.html) (Lupas *et al.*, 1991) for the identification of the coiled coil domain, and ii) by blast searches for conserved domain homologies at the NCBI conserved domain databases (CDD) (http://www.ncbi.nlm.nih.gov/Structure/cdd/wrpsb.cgi) (Marchler-Bauer *et al.*, 2015) for the annotation of the nucleotide biding domain. The annotation of the LRR repeats was done using an in-house script.

The *Pm2* orthologous and homologous genes were identified by BLAST searches against the wheat survey sequence (https://urgi.versailles.inra.fr/blast/blast.php) and the barley high confidence gene database (http://webblast.ipk-gatersleben.de/barley/viroblast.php). Presence of mRNA transcripts indicating gene expression for the *Pm2* homologs in the wheat cultivar 'Chinese Spring' was assessed based on the transcriptome data available at the IWGSC website (https://urgi.versailles.inra.fr/gb2/gbrowse/wheat_survey_sequence_annotation/).

For the AVRPM2 effector family, secretion peptides were predicted using SignalP V4.1 (D-cutoff values 0.5, (Petersen *et al.*, 2011) and disulfide bonds by DISULFIND (Ceroni *et al.*, 2006) after manual reannotation of the genes. Conserved motifs were identified using motif scanning software from the MEME Suite 4.11.2 (Bailey *et al.*, 2009). Sequences corresponding to the predicted mature protein after removal of the predicted signal peptide, and encoded by the *AvrPm2* gene family in wheat and barley powdery mildews were submitted to the RaptorX server for tertiary structure prediction (http://raptorx.uchicago.edu/) (Källberg *et al.*, 2012). Three dimensional protein models were assessed for significant homology to ribonucleases based on the criteria described in Källberg *et al*. (2012).

Notes S1 Sequences of the *Blumeria graminis* genes used for transient expression in *Nicotiana benthamiana.*

(See separate file.)

Notes S2 CDS sequences of the *AvrPm2* family members in *B.g. tritici* and *B.g. hordei.*

(See separate file.)

Notes S3 Protein sequences of the *AvrPm2* family members in *B.g. tritici* and *B.g. hordei.*

(See separate file.)

Notes S4 BAC selection, sequencing and assembly

A BAC (bacterial artificial chromosome) library of the reference isolate 96224 was previously produced (Parlange *et al.*, 2011) and assembled using the fingerprint contig (FPC) algorithm covering each contig with a predicted arrangement of overlapping BACs. The isolate 96224 was then sequenced using the 454 shotgun sequencing technology and the 454 scaffolds were integrated to the BAC assembly using BAC end sequences as linker sequences between 454 scaffolds and FP contigs (Wicker *et al.*, 2013) resulting in genome contigs of the *B.g. tritici* reference genome. In the genetic map, *AvrPm2* is located at 7.0 cM from marker 051_LE. In the *B.g. tritici* reference genome, contig 51 has a size of 2 Mb and contains only few contigs of sequence surrounded by gaps of various lengths (76.4%) (Wicker *et al.*, 2013) which was estimated based on the FPC (Wicker *et al.*, 2013). In our work, we selected five BACs from the BAC library based on the FPC assembly that comprise the complete physical interval of *AvrPm2* including the flanking markers. Prior to sequencing, 20 genetic markers and 38 physical markers designed on the BAC-end sequences were tested by PCR on the selected BACs to validate the overlap. Overlap between the five BACs was confirmed by marker analysis: at least two markers were amplified from each of two consecutive BACs. The flanking markers F2 and F12 delimited a region estimated at 250 kb based on the FPC. The amplification of marker E-5842_F1 on three of the selected BACs confirmed that the gene *BgtE-5842* is located between the flanking markers (Fig. S2). The LTC assembly showed different overlap compared to the FPC assembly: for example, BAC 24i20 (red in Fig S2a) was located before BACs 11j01 and 11o01 in the FPC assembly and after these same BACs in the LTC. The amplification obtained with the PCR markers confirmed that the LTC assembly is better than the FPC: marker 3b20f_F1 showed amplification on four BACs (3k11, 8e01 11o01 and 24i20) but not on 11j01 located between 11o01 and 24i20 (Fig. S2). In the LTC assembly the overlap between these three BACs was reorganized and therefore marker 3b20f_F1 amplified from four neighboring BACs.

The five BACs covering the region between the markers F2 and F12 were completely sequenced and assembled using two different sets of parameters. A permissive assembly was done with all reads obtained by sequencing (on average 2 million per BAC) giving an average of 5.2 contigs per BAC. A stringent assembly was done using 120,000 to 200,000 reads per BAC cleaned from bacterial vector backbone sequences. With the second assembly more and smaller contigs were obtained with an average of 12.2 contigs per BAC. The permissive assembly containing the

bacterial backbone was useful to localize the beginning and the end of each BAC clone sequence. Thus, contigs obtained with the stringent assembly could be ordered by comparing them to the bigger contigs resulting from the permissive assembly (Fig. S3b). Finally, all the assemblies generated for all the BACs were compared and aligned to obtain a contiguous sequence. The model assembly was created using six contigs from four different BACs (Fig. S3c). This assembly was validated by locating the genetic markers on the sequence. The size of the model assembly was 217 kb which is smaller than the originally predicted size (i.e. 250 kb between F2 and F12).

Notes S5 Genetic and physical mapping of *AvrPm2* based on the *B.g. tritici*/*B.g. hordei* genome collinearity

It was previously observed that orthologous genes in *B.g. tritici* and *B.g. hordei* show a strong collinearity as their order and orientation are highly conserved (Oberhaensli *et al.*, 2011; Wicker *et al.*, 2013). We exploited this collinearity to search for homologs of the genes located in the *AvrPm2* region that are present in the *B.g. hordei* genome. In the candidate region of contig 51, nine genes were predicted. Those genes were used in BLAST searches against the *B.g. hordei* genome and homologs of these genes were found on contig 47 of *B.g. hordei*. The genes from *B.g. hordei* were then used in BLAST searches against the *B.g. tritici* genome. We found that three of the nine identified genes from *B.g. hordei* had homologs on contig 48 of *B.g. tritici* which, according to the genome-wide map, is located on the same linkage group as contig 51 (S1 Data). Five *B.g. hordei genes* and their homologs in *B.g. tritici* showed a strong collinearity: both gene order and transcriptional orientation are conserved. *Bgt-2686* was predicted on a sequence island on the right of *Bgt-1444* but its homolog on *B.g. hordei* was situated on the left of *Bgh-3405* (Fig. S2). Markers were designed on this gene and tested by sequencing. It co-segregated with marker F2 at 9 recombinants to *AvrPm2* revealing that the segment of sequence containing *Bgt-2686* was misassembled and could be located in the sequence gap on the F2 side of *AvrPm2* (Fig. 12c). Three of the nine genes in contig 51 were CSEPs from the same gene family (*BgtE-5843*, *BgtE-5845* and *BgtE-5846*). BLAST searches using these effectors resulted in the identification of two homologous CSEPs in *B.g. hordei* (Fig. S2). When used again for BLAST searches against the *B.g. tritici* genome, a new homolog was found in *B.g. tritici*, *BgtE-5842* which was predicted on an unanchored scaffold (Fig. S2). Markers were designed on this gene and tested by sequencing. *BgtE-5842* mapped genetically near the *AvrPm2* locus at 2.0 cM from *AvrPm2*, anchoring the scaffold inside the locus and reducing the genetic interval by 1.9 cM (Fig. 2c).

References

Bailey TL, Boden M, Buske FA, Frith M, Grant CE, Clementi L, Ren J, Li WW, Noble WS. **2009**. MEME SUITE: tools for motif discovery and searching. *Nucleic Acids Research* **37**: W202–W208.

Bourras S, McNally KE, Ben-David R, Parlange F, Roffler S, Praz CR, Oberhaensli S, Menardo F, Stirnweis D, Frenkel Z *et al.* 2015. Multiple avirulence loci and allele-specific effector recognition control the Pm3 race-specific resistance of wheat to powdery mildew. *Plant Cell* **27**: 2991–3012.

Ceroni A, Passerini A, Vullo A, Frasconi P. **2006**. DISULFIND: a disulfide bonding state and cysteine connectivity prediction server. *Nucleic acids research* **34**: W177–81.

Danecek P, Auton A, Abecasis G, Albers CA, Banks E, DePristo MA, Handsaker RE, Lunter G, Marth GT, Sherry ST *et al.* 2011. The variant call format and VCFtools. *Bioinformatics* **27**: 2156–2158.

Edgar RC. **2004**. MUSCLE: multiple sequence alignment with high accuracy and high throughput. *Nucleic Acids Research* **32**: 1792–1797.

Enright AJ, Van Dongen S, Ouzounis CA. **2002**. An efficient algorithm for large-scale detection of protein families. *Nucleic acids research* **30**: 1575–84.

Frenkel Z, Paux E, Mester D, Feuillet C, Korol A, Lander E, Linton L, Birren B, Nusbaum C, Zody M *et al.* 2010. LTC: a novel algorithm to improve the efficiency of contig assembly for physical mapping in complex genomes. *BMC Bioinformatics* **11**: 584.

Källberg M, Wang H, Wang S, Peng J, Wang Z, Lu H, Xu J. **2012**. Template-based protein structure modeling using the RaptorX web server. *Nature Protocols* **7**: 1511–22.

Lupas A, Van Dyke M, Stock J. **1991**. Predicting coiled coils from protein sequences. *Science* **252**: 1162–1164.

Marchler-Bauer A, Derbyshire MK, Gonzales NR, Lu S, Chitsaz F, Geer LY, Geer RC, He J, Gwadz M, Hurwitz DI *et al.* 2015. CDD: NCBI's conserved domain database. *Nucleic Acids Research* **43**: D222–6.

Menardo F, Praz CR, Wyder S, Ben-David R, Bourras S, Matsumae H, McNally KE,

Parlange F, Riba A, Roffler S *et al.* 2016. Hybridization of powdery mildew strains gives rise to pathogens on novel agricultural crop species. *Nature Genetics* **48**: 201–205.

Oberhaensli S, Parlange F, Buchmann JP, Jenny FH, Abbott JC, Burgis TA, Spanu PD, Keller B, Wicker T. **2011**. Comparative sequence analysis of wheat and barley powdery mildew fungi reveals gene colinearity, dates divergence and indicates host-pathogen co-evolution. *Fungal Genetics and Biology* **48**: 327–334.

Parlange F, Oberhaensli S, Breen J, Platzer M, Taudien S, Simková H, Wicker T, Doležel J, Keller B. **2011**. A major invasion of transposable elements accounts for the large size of the *Blumeria graminis* f.sp. *tritici* genome. *Functional & Integrative Genomics* **11**: 671–677.

Pennington HG, Li L, Spanu PD. **2016**. Identification and selection of normalization controls for quantitative transcript analysis in *Blumeria graminis*. *Molecular Plant Pathology* **17**: 625–633.

Petersen TN, Brunak S, von Heijne G, Nielsen H. **2011**. SignalP 4.0: discriminating signal peptides from transmembrane regions. *Nature Methods* **8**: 785–786.

Ristaino JB, Madritch M, Trout CL, Parra G. **1998**. PCR amplification of ribosomal DNA for species identification in the plant pathogen genus *Phytophthora*. *Applied and environmental microbiology* **64**: 948–54.

Wicker T, Oberhaensli S, Parlange F, Buchmann JP, Shatalina M, Roffler S, Ben-David R, Doležel J, Šimková H, Schulze-Lefert P *et al.* 2013. The wheat powdery mildew genome shows the unique evolution of an obligate biotroph. *Nature Genetics* **45**: 1092–1096.

Wu TD, Nacu S. **2010**. Fast and SNP-tolerant detection of complex variants and splicing in short reads. *Bioinformatics (Oxford, England)* **26**: 873–881.
