# Supplementary material for: AvrPm2 encodes an RNase‐like avirulence effector which is conserved in the two different specialized forms of wheat and rye powdery mildew fungus
Source: New Phytol. 2016 Dec 9;213(3):1301–14. doi: 10.1111/nph.14372 (PMC5347869; doi:10.1111/nph.14372)
Supplement: Supplementary file 7 [file NPH-213-1301-s007.docx]

**Fig. S8** Annotated protein alignment of the AVRPM2 effector family in *B. g. tritici* and *B. g. hordei*.

**Table S9** Blast hits of PM2 against wheat and barley databases

**Table S12** Signal peptide and disulphide bond predictions in the AVRPM2 family

**Table S13** Structure prediction of the AVRPM2 in *B. g. tritici* and *B. g. hordei*

**Table S8** Genetic map of the 96224 9 JIW2 mapping population based on KASP markers

**Notes S3** Protein sequences encoded by *AvrPm2* family members in *B. g. tritici* and *B. g. hordei*.
